# Nuclear factor erythroid 2‐related factor 2 ameliorates disordered glucose and lipid metabolism in liver: Involvement of gasdermin D in regulating pyroptosis

**DOI:** 10.1002/ctm2.70233

**Published:** 2025-02-24

**Authors:** Xuyun Xia, Qin Zhang, Xia Fang, Ling Li, Gangyi Yang, Xiaohui Xu, Mengliu Yang

**Affiliations:** ^1^ Department of Endocrinology, The Second Affiliated Hospital Chongqing Medical University Chongqing China; ^2^ The Key Laboratory of Laboratory Medical Diagnostics in the Ministry of Education and Department of Clinical Biochemistry, College of Laboratory Medicine Chongqing Medical University Chongqing China; ^3^ College of Stomatology Chongqing Medical University Chongqing China

**Keywords:** glucose/lipid metabolism, GSDMD, NRF2, pyroptosis

## Abstract

**Background:**

The epidemic of metabolic dysfunction‐associated fatty liver disease linked to excessive high‐fat diet (HFD) consumption has sparked widespread public concern. Nuclear factor erythroid 2‐related factor 2 (NRF2) has been reported to improve glucose/lipid metabolism, liver lipid degeneration and alleviate HFD‐induced inflammation. However, its pathways and mechanisms of action are not fully understood.

**Methods:**

To confirm the effect of NRF2 on glucose/lipid metabolism in the liver, Nrf2‐/‐ mice as well as liver‐specific Nrf2 knockout mice, and AAV‐TBG‐Nrf2 were employed. The hyperinsulinemic‐euglycemic clamp was utilized to determine the effect of NRF2 on glucose metabolism. To elucidate the effect of NRF2 on pyroptosis, we performed western blots, immunofluorescence, quantitative real‐time PCR, and Flow cytometry experiments. Finally, chromatin immunoprecipitation‐seq and dual‐luciferase reporter assay was used to underscore the transcriptional regulatory effect of NRF2 on Gsdmd.

**Results:**

We found that overexpression of Nrf2 inhibited the expression of inflammatory cytokines and pyroptosis markers, including cle‐Caspase1, NLRP3 and the N‐terminus of gasdermin D (N‐GSDMD) both in vivo and in vitro, while Nrf2 deficiency was the opposite. Specifically, with NRF2 expression up‐regulated, GSDMD expression decreased and Gsdmd overexpression partially reversed the effect of Nrf2 overexpression on pro‐inflammatory phenotype. Mechanistically, we demonstrate that NRF2 binds to the Gsdmd promoter at the −2110 ‐ 1130 bp site, inhibiting the GSDMD expression and thereby improving glucose/lipid metabolism and liver steatosis.

**Conclusion:**

Our data indicate that NRF2 is an effective inhibitor of pyroptosis and has a multi‐target effect in the treatment of obesity‐related metabolic diseases.

**Key points:**

- MAFLD is associated with increased hepatocytes NRF2 expression.
- NRF2 alleviates MAFLD by suppressing pyroptosis.
- NRF2 directly inhibits GSDMD expression to regulate pyroptosis.
- Targeting the NRF2–pyroptosis (GSDMD) axis offers a potential therapeutic strategy for MAFLD.

## Graphical abstract

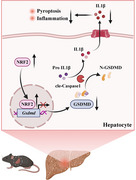

## 1 INTRODUCTION

The escalating incidence of metabolic dysfunction‐associated fatty liver disease (MAFLD), associated with elevated energy intake and reduced physical activity, has raised significant public health concerns. The pathogenesis of MAFLD is multifactorial, involving insulin resistance (IR), oxidative stress and inflammation,^1^ leading to increased hepatocellular death. While apoptosis and necrosis were traditionally emphasised in MAFLD, recently research highlights the role of other programmed cell death, such as necroptosis and pyroptosis.^2^ In pyroptosis pathways, the Nod‐like receptor (NLR) Pyrin domain 3 (NLRP3) initiate the activation of caspases‐1, which leaves gasdermin D (GSDMD) to produce gasdermin‐N domain (N‐GSDMD) and also processes pro‐IL1β and IL18 into their active forms.^3^ GSDMD is a key executor of pyroptosis, upon cleavage, the N‐GSDMD triggers the form of pores in the cell membrane, facilitating the release of IL1β and IL18, which amplify inflammatory responses.^4^, ^5^ While pyroptosis aids in clearing pathogens, excessive pyroptosis can cause tissue damage and organ failure.^6^, ^7^ Recent studies have linked pyroptosis to the progression of MAFLD, associating excessive lipid deposition with increased inflammation and fibrosis. Inhibiting pyroptosis in hepatocytes has been shown to mitigate liver damage, highlighting pyroptosis as a potential therapeutic target for MAFLD.^4^, ^8^ A thorough understanding of related mechanisms is crucial for developing effective treatments.

Nuclear factor erythroid 2‐related factor 2 (NRF2) has emerged as a critical regulator in the pathophysiology of MAFLD due to its pivotal role in defensing against oxidative stress and inflammation.^9^, ^10^, ^11^ Recent studies have begun to elucidate the interplay between NRF2 and pyroptosis in various tissues. Evidence suggest that NRF2 inhibits pyroptosis in microglia and cardiomyocytes,^12^, ^13^ suggesting that NRF2 may play a broader role in preventing pyroptosis. Since reactive oxygen species critically trigger the activity of NLRP3 and stabilise N‐GSDMD‐mediated pore formation,^14^ the anti‐oxidative response mediated by NRF2 is believed to underlie its inhibitory effect on pyroptosis. However, the exact role and molecular mechanism of NRF2 in high‐fat diet (HFD)‐induced hepatic pyroptosis remain unknown.

Herein, we identified that NRF2 suppresses hepatocyte pyroptosis in the liver, resulting alleviated liver inflammation caused by a HFD. The down‐regulation of GSDMD expression by NRF2 is involved in its positive role against MAFLD. We found that NRF2 directly suppresses GSDMD transcription expression through chromatin immunoprecipitation (ChIP) and dual‐luciferase reporter assay. Our results provide a more comprehensive view of the mechanisms regarding the regulatory role of NRF2 in pyroptosis and underscore its significance in MAFLD.

## 2 MATERIALS AND METHODS

### 2.1 Human samples

Liver tissues were collected through hepatectomy from patients with liver tumours at the outpatient clinic of the Second Affiliated Hospital of Chongqing Medical University. Non‐tumourous liver regions were isolated and classified as either MAFLD (*n* = 4) or control (*n* = 4) based on histological examination. Individuals diagnosed with viral hepatitis, diabetes or other communicable diseases were excluded. Informed consent was obtained from all subjects, and the study was ethically approved (2021‐156) by The Second Affiliated Hospital of Chongqing Medical University.

### 2.2 Animals

Five‐week‐old male C57BL/6J mice (WT mice) were purchased from Gempharmatech Co (Nanjing, China) and adapted for 3 weeks before starting HFD‐feeding to establish the model. db/db mice were acquired from Cyagen (Suzhou, China). *Nrf2^−/−^* mice were generously provided by Dr Ni Tang (Chongqing Medical University) and these mice were generated and identified as previously reported.^15^ *Nrf2^fl/fl^* mice were purchased from Cyagen (Suzhou, China). All mice were housed at Chongqing Medical University's animal centre under a 12‐h light/dark cycle at 22 ± 2°C. Eight‐week‐old mice were fed ad libitum to either a normal chow diet (ND) (4% calories from fat; Keao Xieli Feed Co., Ltd., China) or a HFD (60% calories from fat; Research Diets, USA) as indicated.

To establish liver‐specific *Nrf2* knockout (*Nrf2^LKO^*) mice, 8‐week‐old male *Nrf2^fl/fl^* mice received an adeno‐associated virus with thyroxine‐binding globulin (TBG) expressing *Cre/*Null (AAV8–TBG–*Cre/*Null, 5 × 10^11^ vg/mouse) injection via tail vein. After recovery for 2 weeks, the mice were given a methionine/choline deficiency (MCD) diet (A02082002BR; Research Diets) for 4 weeks. For mice liver‐specific *Nrf2* overexpression, 8‐week‐old male C57BL/6J mice received a tail vein injection of adeno‐associated virus with TBG expressing *Nrf2* or GFP (AAV8–TBG–*Nrf2/*GFP, 8 × 10^11^ vg/mouse), followed by HFD feeding for 12 weeks. For *Gsdmd* knockdown in *Nrf2* ^−/−^ mice, 8‐week‐old *Nrf2^−/−^* mice were tail vein injected with lentivirus expressing *shGsdmd* or control (Lv‐*shGsdmd/*Con, 5 × 10^7^ TU/mouse) and fed a HFD for 8 weeks. The sequence of *sh*RNA targeting *Gsdmd* was 5′‐GCAGCATGAAAGGCACCTTCA‐3′. The efficiency of the knockdown was assessed by WB and PCR. All animal studies received approval from the Animal Experimentation Ethics Committees of Chongqing Medical University (IACUC‐CQMU‐2024‐0083).

### 2.3 Glucose and insulin tolerance tests

On the 12th weekend of ND or HFD feeding, mice were fasted for 14–16 h for glucose tolerance test (GTT) and 4–6 h for insulin tolerance test (ITT). Mice were given an intraperitoneal injection of 20% glucose (1 g/kg; KELUN, China) or insulin (0.75 U/kg; Novo Nordisk, Denmark). Blood samples were obtained from tail bleeds and were subsequently measured with a glucometer (OneTouch Verio®; Johnson & Johnson, USA) at various time points: at baseline, as well as 15, 30, 60 and 120 min post‐injection.

### 2.4 Hyperinsulinemic‐euglycemic clamps

Hyperinsulinemic‐euglycemic clamps were performed as previously described.^16^ In brief, mice underwent a 10 h fasting period prior to the study, after which they were anesthetised with isoflurane anesthesia (induction 5% and maintenance 2%). [3‐^3^H]‐glucose (0.2 mL/h; 0.05 µCi/min) was continuously infused through the jugular vein for 90 min and maintained throughout the study. At *t* = 0 min, insulin (5 mU/kg min) and 20% glucose were infused into the jugular vein and maintained throughout the study. Samples of blood were collected at −90, 0, 80, 90, 100 and 120 min to assess the specific activities of [3‐^3^H]‐glucose.

### 2.5 Cell culture

Mouse primary hepatocytes (MPHs) were extracted from WT and *Nrf2^−/‐^* mice, as previously reported.^17^ MPHs and HepG2 cells were cultured in DMEM with 10% FBS and 1% penicillin–streptomycin–amphotericin B, maintained at 37°C in a 5% CO₂ incubator. For adenovirus (Adv)‐induced *Nrf2* overexpression, HepG2 cells were transfected with Adv‐*Nrf2/*Con (OBiO Inc, Shanghai, China) for 24 h. For *Gsdmd* overexpression or knockdown, HepG2 cells were transfected with Lv‐*Gsdmd/shGsdmd/*Con for 48 h. Palmitic acid (PA) solution was prepared by adding 33 mg PA (P5585; Sigma) into 3 mL of 0.1 M NaOH, and then incubated at 75°C until completely dissolved. To prepare the BSA solution, 1.2 g of free fatty acids were first dissolved in 3 mL of PBS. Afterward, the PA solution was mixed into the BSA solution, which was subsequently filtered. The mixture was stored at −20°C. To construct a cell model of lipid metabolism disorder, HepG2 cells or MPHs were treated with 50–800 µM PA for 24 h.

### 2.6 Western blots

After sacrificing the mice, liver samples were collected and initially homogenised in RIPA Lysis Buffer (Beyotime, China; P0013B) supplemented with phenylmethanesulfonyl fluoride (Beyotime; ST506), using a tissue grinding machine equipped with steel balls (JXFSTPRP‐CL, Jingxin, China). Following centrifuging (12 000×*g*, 15 min), the supernatant underwent sonication and centrifugation (12 000×*g*, 10 min). Protein level of the resulting supernatant was quantified using BCA detecting method (Beyotime; P0010S). After being separated by SDS‐PAGE, the proteins were transferred onto Immobilon‐P PVDF membranes. Subsequently, immunoblotting was conducted using primary and secondary antibodies, detailed in Table S1.

### 2.7 Immunofluorescence

After fixing the cells in 4% paraformaldehyde for 30 min, they were permeabilised with 0.3% Triton X‐100. To avoid nonspecific binding, the cells were incubated with 10% goat serum at room temperature for 1 h. Subsequently, the cells were treated with anti‐GSDMD at 4°C for 16 h, followed by a 2‐h treatment with goat anti‐mouse IgG H&L at 37°C. Nucleus were stained with DAPI (C1002; Beyotime). Fluorescent images was obtained by a confocal microscopy (TSC SP8; Leica, Germany) using 20× and 40× objectives. Images were processed using the LAX V Version 1.0.0 build 0 and ImageJ (NIH; ver. 1.52t.) software.

For immunofluorescence (IF) staining in the liver, tissues were fixed and dehydrated (30% sucrose diluted in 4% paraformaldehyde) for 48 h, then sectioned into 6 µm slices. The followed IF staining procedures were the same as described above. The antibodies for IF staining were summarised in Table S1.

### 2.8 Quantitative real‐time PCR

Total RNA was extracted using RNAiso Plus (9109; Takara, Japan), and its quality and concentration were assessed with a Nanodrop 2000 spectrophotometer. Complementary DNA (cDNA) was generated from 0.5 µg of RNA with the PrimeScript™ RT Reagent Kit (RR036A; Takara), and qRT‐PCR was subsequently conducted on the CFX Opus 96 Real‐Time qPCR system. Briefly, the reaction consists of a 10 µL system including 5.6 µL TBgreen (RR420A; Takara), 10 pM primer and 2 µL cDNA. A two‐step cycling protocol was performed as follows: initial denaturation at 95°C for 30 s, followed by 40 cycles of 95°C for 5 s and 60°C for 30 s. Melt curve analysis was conducted from 65 to 95°C with a 0.5°C increment per cycle. Primer sequences are listed in Table S2.

### 2.9 TUNEL assay

Mice liver tissues were fixed overnight in 4% paraformaldehyde, then sectioned into 6 µm slices. Cell death was detected using either a fluorescein (G1501‐50T; Servicebio, China) or a TMR (G1502‐50T; Servicebio) TUNEL cell apoptosis detection kit. Briefly, the slices of tissues were treated with proteinase K solution for 10 min, followed by a 10‐min incubation with equilibration buffer. TdT buffer was then used for incubation of the slices at 37°C for 1 h. Images were captured using a confocal microscopy.

### 2.10 Histological staining

Mice liver were paraformaldehyde fixed and paraffin embedded, and sectioned at 6 µm. For H&E staining, haematoxylin and eosin (Servicebio) were applied and incubated for 3–5 min. Oil‐Red‐O solution (G1015; Servicebio) was utilised to stain frozen liver sections or HepG2 cells for 30 min to measure lipid deposition. For Sirius red staining, sections first underwent deparaffinisation and rehydration. Sirius red (PH1098; PHYGENE, China) was utilised to incubate sections for 1 h and followed by differentiation with acidified water. After dehydration with 100% ethanol and xylene, the sections were imaged with a slide scanner (VS200; Olympus, Japan). Frozen liver tissue sections were stained with 2 µM BODIPY493/503 (D3922; Invitrogen, USA) for 30 min for BODIPY staining. The image was obtained by fluorescence microscopy.

### 2.11 Cell counting kit‐8 assay

MPHs or HepG2 cells plated with 96‐well culture plates were administrated with PA (50, 100, 200, 400 and 800 µM) for a 24‐h period. The medium was then refreshed and cell counting kit‐8 (CCK8) solution (C0038; Beyotime) was added, followed by incubation at 37°C for 1 h. Finally, A450 was measured using a Spectramax iD5 microplate reader (Molecular Devices, USA).

### 2.12 Enzyme‐linked immunosorbent assay and lactate dehydrogenase assay

HepG2 cells or MPHs were seeded into six‐well culture plates (6 × 10^5^/well). After a 24‐h transfection with Adv‐*Nrf2*/Con, HepG2 cells were treated with Lv‐*Gsdmd*/Lv‐Con for 48 h, and then subjected to 200 µM PA for 24 h. Following the treatment, the supernatant was collected and centrifuged at 1000×*g* for 5 min. IL1β concentrations were measured using a human enzyme‐linked immunosorbent assay (ELISA) kit (RX106152H; Ruixin Biotech, China) or a mouse ELISA kit (orb775136; Biorbyt, UK). Lactate dehydrogenase (LDH) activity was assessed by using a commercial kit (C0016; Beyotime). Briefly, HepG2 cells or MPHs were treated similarly to ELISA experiments. Following a 5‐min centrifugation at 400×*g*, 120 µL of the supernatant was carefully collected. Sixty microliters of LDH solution was added and then incubated at room temperature for 30 min. The A490 was measured using a microplate reader.

### 2.13 Flow cytometry

The APC Annexin V/FITC PI Kit was employed to assess cell pyroptosis levels. Cells were briefly washed and resuspended in PBS, stained with APC Annexin V and PI at 25°C in the dark for 15 min and analysed using a flow cytometer (Beckman Coulter, USA) within an hour, with subsequently analysed by FlowJo software (v10.6.2).

### 2.14 Prediction of the relationship between NRF2 and GSDMD

To identify potential NRF2 binding sites within the promoters of pyroptosis‐related genes, the JASPAR database (https://jaspar.elixir.no/) was utilised. Relative profile scores for NRF2 binding motifs in target genes were compared. Additionally, the GEPIA database (http://gepia.cancer‐pku.cn/) was used to assess the correlation between NRF2 and the mRNA expression levels of pyroptosis markers.

### 2.15 Dual‐luciferase reporter assay

A luciferase reporter plasmid (pGL4.1–*Gsdmd*–WT) was constructed with the full‐length human *Gsdmd* promoter. Two truncated *Gsdmd* promoters, −2110 to −1130 bp (*Gsdmd*#1) and −1129 to 0 bp (*Gsdmd*#2), were also cloned into the pGL4.1 plasmid. HepG2 cells were co‐transfected with pGL4.1–*Gsdmd*–WT/*Gsdmd*#1/*Gsdmd*#2, PGL3.1–*Nrf2*/Con and Renilla luciferase reporter plasmid (pRL‐CMV) by using Lipofectamine 3000 (L3000015; Thermo Fisher Scientific, USA) for 48 h. The Dual‐Lumi™ II Luciferase Reporter Gene Assay Kit (RG089S; Beyotime) was used to assess the signals of firefly and Renilla luciferase. Firefly luciferase results were normalised against Renilla luciferase activity.

### 2.16 Chromatic immunoprecipitation

The ChIP assay was conducted using 250 000 HepG2 cells or 1 mg of lightly fixed liver tissue from mice transfected with AAV–TBG–*Nrf2* with the CUT&RUN assay kit (Cell Signaling Technology; #86652S), as previously reported.^18^ Briefly, cultured HepG2 cells or single‐cell suspension from liver tissue pieces were washed and incubated with concanavalin A‐beads for 5 min. After washing, the cell‐bead solution was further incubated with 4 µg anti‐NRF2 antibody at 4°C for 4 ‐ 6 h. Following this, 50 µL of pAG‐MNase pre‐mix was added to each tube and incubated at 4°C for 1 h. After washing, 3 µL cold CaCl_2_ was added to activate pAG‐MNase. Then, 1× stopping buffer was added to release DNA fragments and purification. The purified DNA products were quantified by qPCR and NG‐Sequencing. For constructing sequencing libraries, CUT&RUN DNA was processed with a NEBNext Ultra II DNA Library Prep Kit.

To confirm NRF2's targeting of the *Gsdmd* promoter, the CUT&RUN assay was performed, and RT‐PCR was employed to quantify immunoprecipitated DNA by using primers sequence for the *Gsdmd*#1 promoter (forward: CTCCATCCTGGTGACAGAGCA; reverse: GCACTCCTGGGCTCAAGTGATC) and *Gsdmd*#2 promoter (forward: GATGC TGCCGTGAACTTTGGT; reverse: TCTGGAAT AAGCCTGGCAGCT).

### 2.17 Statistical analysis

The data were expressed as means ± SEM. An unpaired two‐tailed Student's *t*‐test was used for two‐group comparisons. For more than two groups, one‐way ANOVA was used, with Tukey's post hoc test for multiple comparisons. GraphPad Prism 9.5.1 was used for the statistical analyses, with a significance threshold of *p* less than .05.

## 3 RESULTS

### 3.1 MAFLD is associated with increased NRF2 expression and pyroptosis in hepatocytes

**FIGURE 1:**
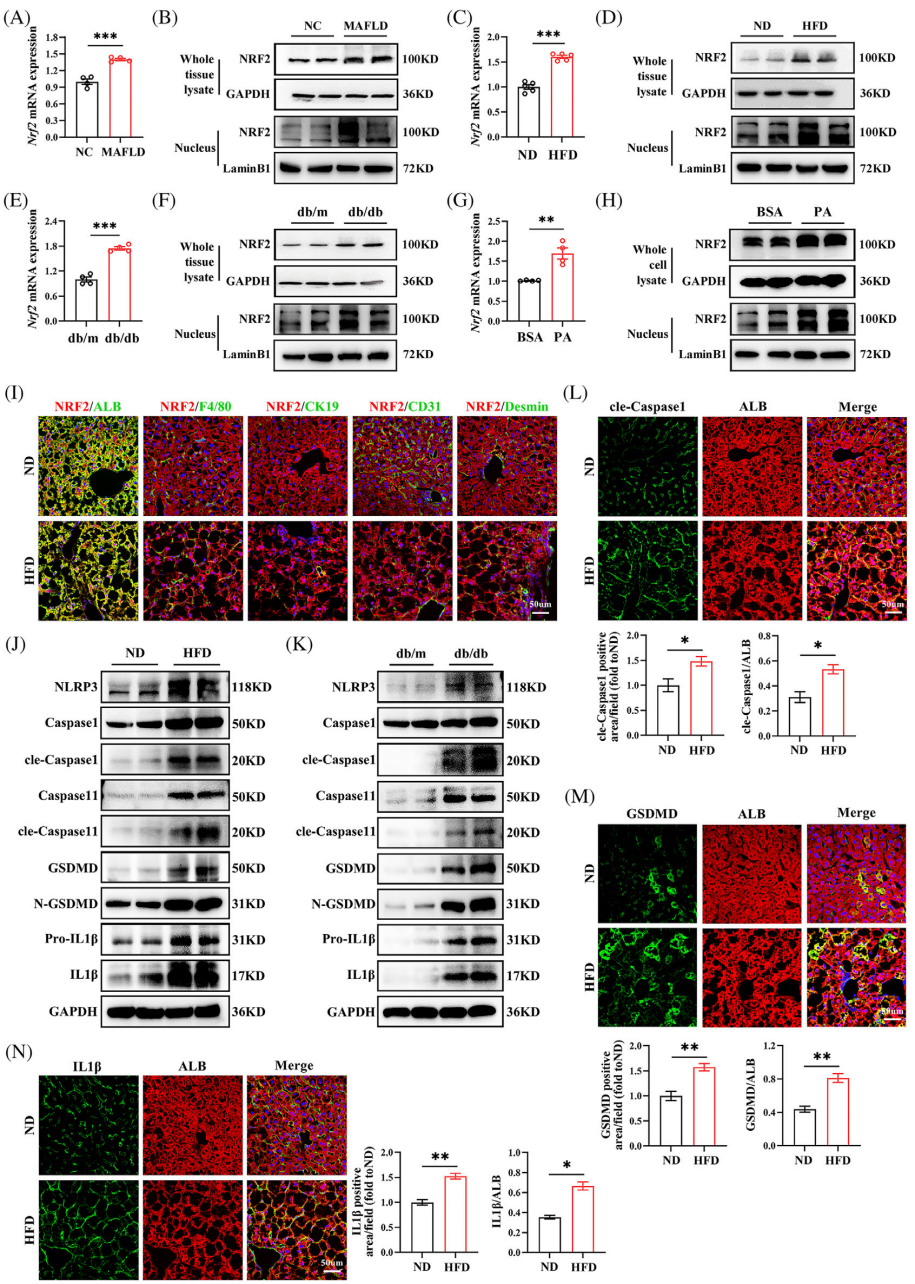
NRF2 expression and pyroptosis are increased in liver of patients with MAFLD and obese mice. (A and B) NRF2 mRNA (A) and protein expression of whole cell lysate and nucleus (B) in normal individuals and patients with MAFLD. (C and D) Eight‐week‐old male WT mice were fed a ND or HFD for 12 weeks. NRF2 mRNA (C) and protein (D) expression in the liver. (E and F) NRF2 mRNA (E) and protein (F) expression in the liver of db/m and db/db mice. (G and H) NRF2 mRNA (G) and protein (H) expression in BSA or PA‐treated HepG2 cells. (I) Co‐immunofluorescence (Co‐IF) for NRF2 with ALB, F4/80, cytokeratin 19 (CK19), CD31 or Desmin. (J and K) Western blots analysis for pyroptotic cell markers in the liver of ND‐ or HFD‐fed WT mice (J) and db/m or db/db mice (K). (L–N) Co‐IF staining for cle‐Caspase1 (L), GSDMD (M) or IL1β (N) with ALB in the liver from ND‐ or HFD‐fed mice. NC, normal control; ND, normal chow diet; HFD, high‐fat diet; MAFLD, Metabolic dysfunction‐associated fatty liver disease; BSA, bovine serum albumin; PA, palmitic acid. Data are expressed as the mean ± SEM (*n* = 3 mice and three independent experiments). *p < .05, **p < .01, ***p < .001.

To determine NRF2's potential involvement in MAFLD, we first analysed its expression in liver tissues from MAFLD patients. Clinical parameters in MAFLD patients are displayed in Table S3. Consistent with previous study,^19^ we found that both NRF2 mRNA and protein levels were significantly elevated in the liver of MAFLD patients compared with controls, along with increased nuclear NRF2 expression (Figures 1A,B and S1A). This increase in NRF2 expression was further corroborated in animal models; specifically, HFD‐fed mice and db/db mice exhibited markedly higher levels of total and nuclear NRF2 expression compared with their counterparts (Figures 1C–F and S1B,C). Additionally, at the cellular level, NRF2 mRNA and protein expression significantly increased in HepG2 cells following PA treatment and increased nuclear NRF2 expression relative to BSA‐treated cells (Figures 1G,H and S1D). To identify the main cell types expressing NRF2 in the liver, we performed Co‐IF staining of NRF2 with liver parenchymal cell markers, including hepatocytes (ALB), macrophages (F4/80), biliary epithelial cells (CK19), sinusoidal endothelial cells (CD31) and hepatic stellate cells (Desmin). We found that NRF2 was mainly co‐localised with ALB, indicating that NRF2 is mainly expressed in hepatocytes. Therefore, HFD mainly leads to an increase in NRF2 expression in hepatocytes (Figure 1I). Overall, these findings suggest that NRF2 is mainly expressed in hepatocytes and closely related to hepatic lipid accumulation.

Given the pivotal role of pyroptosis, a form of programmed inflammatory cell death, in MAFLD pathogenesis,^7^, ^20^ we explored the relationship between MAFLD and hepatic pyroptosis. Our analysis revealed elevated protein levels of key pyroptosis markers, including NLRP3, Caspase1, GSDMD and pro‐IL1β, in the livers of HFD‐fed mice and db/db mice. The levels of cle‐Caspase1, N‐GSDMD and IL1β proteins, which are active pyroptosis markers, significantly increased (Figures 1J,K and S1E,F). IF staining further substantiated these findings, showing significantly higher levels of cle‐Caspase1, GSDMD and IL1β in the hepatocytes of HFD‐fed mice (Figure 1L–N). These data indicate that hepatic steatosis exacerbates hepatic pyroptosis, contributing to MAFLD progression.

### 3.2 Nrf2 knockout aggravates HFD‐induced hepatocyte pyroptosis

**FIGURE 2:**
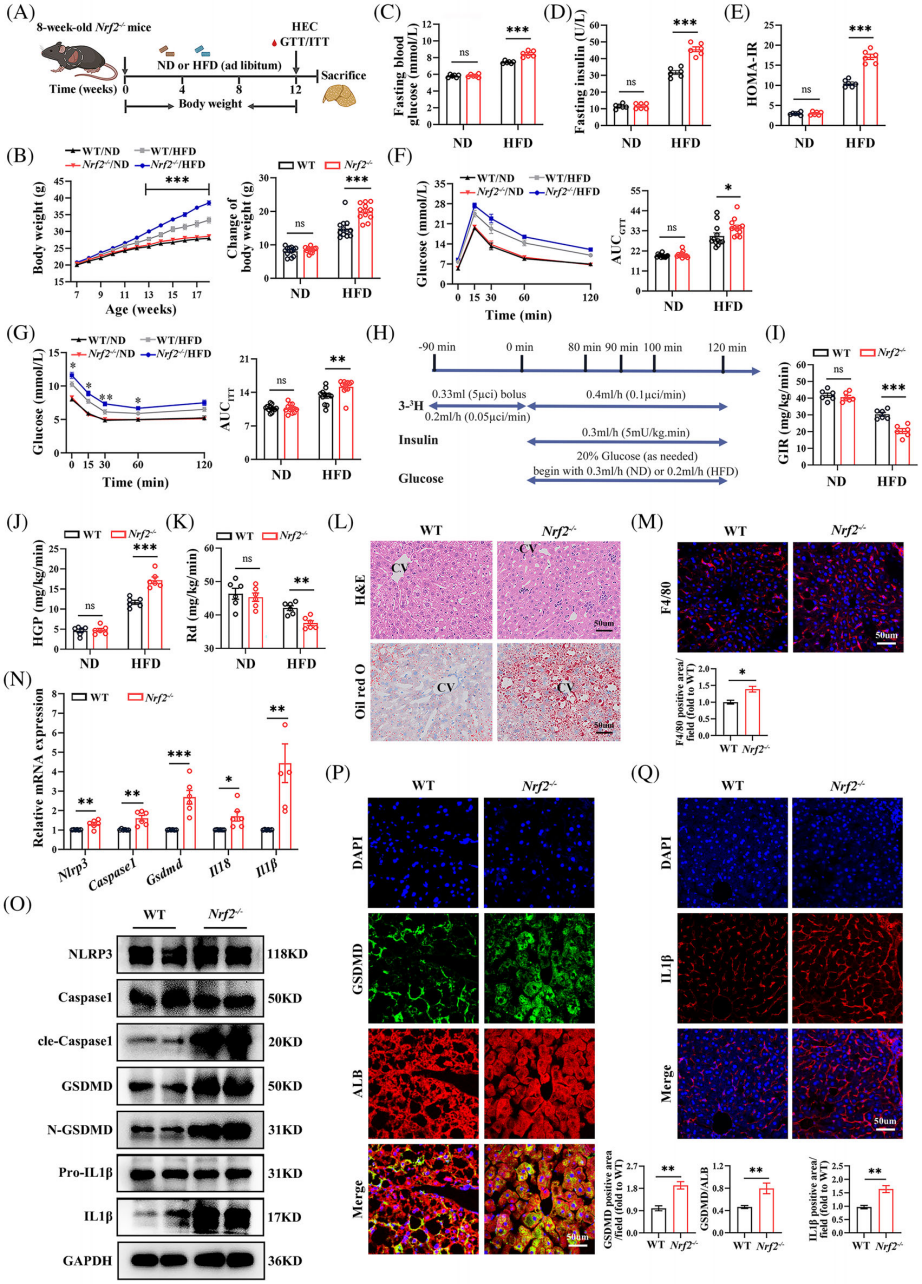
*Nrf2^−/−^* mice exhibit increased body weight, IR, hepatocyte pyroptosis and inflammation under HFD‐feeding. (A) Schematic diagram of the experimental procedure. Eight‐week‐old male WT and *Nrf2^−/−^* mice were fed a ND or HFD for 12 weeks. (B–G) (B) Body weight changes. (C) Fasting blood glucose. (D) Fasting insulin levels. (E) HOMA‐IR. (F) GTT. (G) ITT. (H) Flow chart of hyperinsulinemic‐euglycemic clamps. (I) GIR. (J) HGP. (K) Rd. (L) Hepatic H&E and Oil‐red‐O staining. CV, central vein. (M) IF staining for F4/80 in the liver. (N) Hepatic mRNA expression for pyroptotic cell markers. (O) Western blots analysis for pyroptotic cell markers in the liver. (P) Co‐IF staining of GSDMD and ALB in the liver. (Q) IF staining for IL1β. ND, normal chow diet; HFD, high‐fat diet; GTT, glucose tolerance test; ITT, insulin tolerance test; AUC, Areas under the glucose curves; HOMA‐IR, homeostasis model assessment‐insulin resistance index; GIR, glucose infusion rate; HGP, hepatic glucose production; Rd, glucose disposal. Data are expressed as mean ± SEM (*n* = at least 6 mice and three independent experiments). ns, not significant. *p < .05, **p < .01, ***p < .001.

To investigate the metabolic phenotype associated with NRF2 functional deficiency, we utilised *Nrf2* global knockout (*Nrf2^−/−^*) mice (Figures 2A and S2A). As expected, the expression of NRF2 mRNA and protein in *Nrf2^−/−^* mice was almost invisible (Figure S2B,C). When subjected to a HFD, there was a marked increase in body weight, fasting blood glucose, insulin levels and HOMA‐IR in *Nrf2^−/‐^* mice compared with WT mice. (Figure 2B–E). GTT and ITT further demonstrated impaired glucose tolerance and glucose clearance rates in HFD‐fed *Nrf2^−/−^* mice compared with the WT controls under the same diet (Figure 2F,G). Additionally, EHC experiments (Figure 2H), considered the gold standard for evaluating IR, showed that *Nrf2* deficiency led to decreased glucose infusion rate (GIR), increased hepatic glucose production (HGP) and reduced glucose disposal (Rd) under HFD feeding (Figure 2I–K). The data imply that *Nrf2* deficiency intensifies the metabolic disturbances and IR induced by a HFD.

To further elucidate the role of NRF2 in liver lipid metabolism, we observed the lipid metabolism phenotype in *Nrf2^−/‐^* mice. *Nrf2* knockout mice exhibited increased liver steatosis and chronic inflammatory response, as evidenced by H&E, Oil‐Red‐O, F4/80 and Bodipy staining and increased liver TG content compared with WT mice under HFD‐feeding (Figures 2L,M and S2D,E). However, there was no significant difference in serum ALT and AST (Figure S2F,G), which might be due to HFD not being sufficient to induce marked ALT/AST alterations. Additionally, HFD‐fed *Nrf2^−/−^* mice displayed increased liver fibrosis, indicated by Sirius red and α‐SMA staining (Figure S2H). Consistently, *Nrf2* knockout resulted in increased expressions of genes associated with gluconeogenesis and fat formation, while genes related to glycolysis remained unchanged (Figure S2I–K). These observations suggest that *Nrf2* deficiency exacerbates HFD‐induced hepatic steatosis and chronic inflammation.

Previous studies have shown that NRF2 inhibits pyroptosis in various tissues.^12^, ^13^ However, it remains unclear whether NRF2 plays a role in HFD‐induced hepatocyte pyroptosis. To address this, we measured the mRNA and protein expression of the pyroptosis markers. *Nrf2* knockout led to increased mRNA expression of *Nlrp3*, *Caspase1*, *Gsdmd*, *Il18* and *Il1β* (Figure 2N), as well as elevated protein levels of GSDMD, cle‐Caspase1, N‐GSDMD, pro‐IL1β and IL1β (Figures 2O and S2L). IF staining for cle‐Caspase1 and IL1β, as well as co‐stained GSDMD with ALB, further confirmed the increased hepatocyte pyroptosis in *Nrf2^−/−^* mice (Figures 2P,Q and S2M). TUNEL staining showed an increase in overall cell death in HFD‐fed *Nrf2^−/−^* mice, which includes pyroptosis (Figure S2N). These results demonstrate that *Nrf2* deficiency exacerbates HFD‐induced liver pyroptosis.

### 3.3 Liver‐specific *Nrf2* knockout in mice aggravates MCD‐induced liver hepatocyte pyroptosis

**FIGURE 3:**
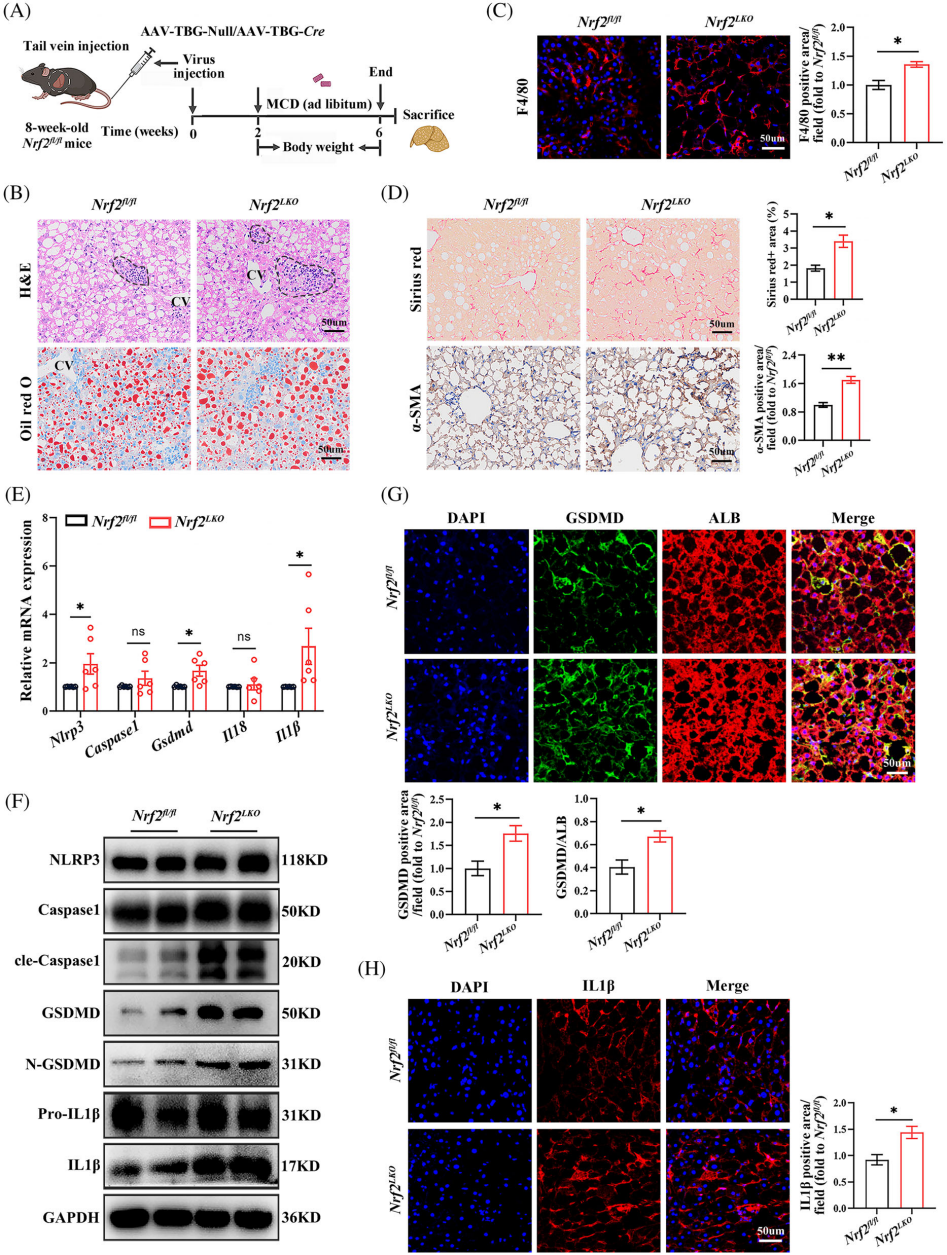
Liver‐specific *Nrf2* knockout aggravates MCD‐induced lipid deposition, inflammation and hepatocyte pyroptosis in the liver. (A) Schematic diagram of the experimental procedure. Eight‐week‐old male *Nrf2^fl/fl^* mice were injected with AAV8–TBG–*Cre*/Null via the tail vein and fed with MCD for 4 weeks. (B) Hepatic H&E and Oil‐red‐O staining. CV, central vein. (C) IF staining for F4/80 in the liver. (D) Sirius red staining and immunohistochemistry staining for α‐SMA in the liver. (E) Hepatic mRNA expression for pyroptotic cell markers. (F) Western blots analysis for pyroptotic cell markers in the liver. (G) Co‐IF staining for GSDMD and ALB. (H) IF staining for IL1β. MCD, methionine and choline‐deficient; α‐SMA, alpha‐smooth muscle actin. Data are expressed as mean ± SEM (*n* = at least 6 mice and three independent experiments). ns, not significant. *p < .05, **p < .01.

To examine NRF2's impact on pyroptosis in hepatocytes, we generated *Nrf2^LKO^* mice and *Nrf2^fl/fl^* mice were used as controls (Figure S3A). These mice were then fed a MCD diet for 4 weeks (Figure 3A). The genotype of the mouse was identified, and the efficiency of *Nrf2* knockout was confirmed by NRF2 mRNA and protein expression (Figure S3B,C). *Nrf2^LKO^* mice exhibited body weights comparable to the *Nrf2^fl/fl^* controls, but showed increased hepatic lipid deposition and macrophage infiltration (Figures 3B,C and S3D,E). Therefore, we believe that the body weight phenotype of *Nrf2^−/−^* mice is caused by the extrahepatic effects of NRF2. Additionally, liver fibrosis was exacerbated in *Nrf2^LKO^* mice (Figure 3D). Consistent with these findings, genes associated with gluconeogenesis and lipid metabolism were up‐regulated in *Nrf2^LKO^* mice compared with controls (Figure S3F–H), indicating that liver‐specific *Nrf2* deletion promotes lipid accumulation and fibrosis in response to the MCD diet.

Next, we examined the impact of liver‐specific *Nrf2* knockout on pyroptosis. As anticipated, *Nrf2^LKO^* mice exhibited elevated expression of pyroptosis markers. This was evidenced by increased mRNA levels of *Nlrp3*, *Gsdmd* and *Il1β*, along with higher protein expression of cle‐Caspase1, N‐GSDMD, GSDMD and IL1β (Figures 3E,F and S3I). IF staining further confirmed these findings, showing increased levels of cle‐Caspase1 and IL1β, as well as increased co‐localisation of GSDMD with ALB in the liver (Figures 3G,H and S3J). Tunnel staining showed the overall cell death in liver of *Nrf2^LKO^* mice (Figure S3K). These results suggest that liver‐specific *Nrf2* knockout exacerbates hepatocyte pyroptosis in response to MCD diet‐induced metabolic stress.

### 3.4 Liver‐specific *Nrf2* overexpression alleviates HFD‐induced liver hepatocyte pyroptosis

**FIGURE 4:**
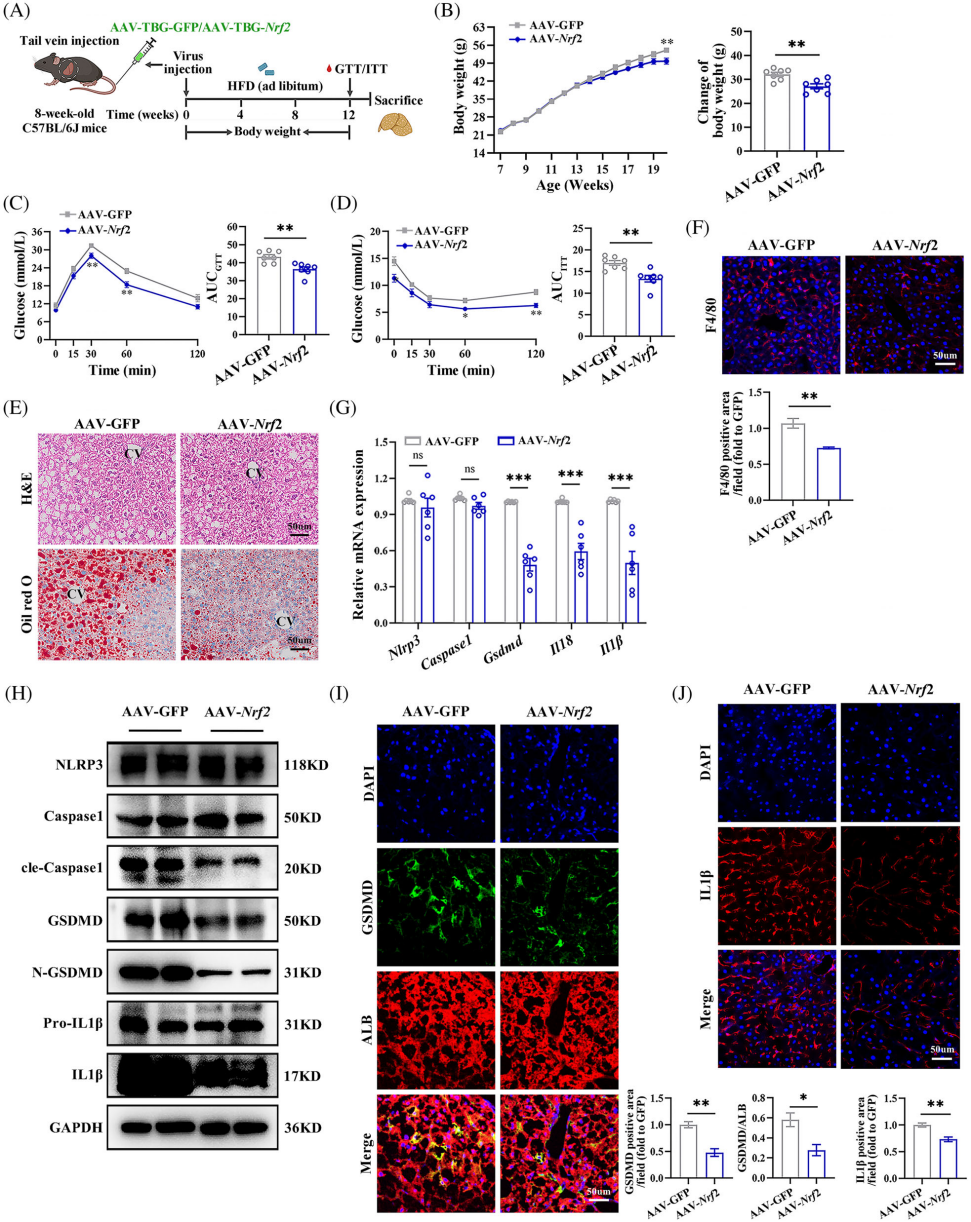
Liver‐specific *Nrf2* overexpression alleviates diet‐induced obesity (DIO), IR, inflammation and hepatocyte pyroptosis in the liver. (A) Schematic diagram of the experimental procedure. Eight‐week‐old male WT mice were injected with AAV8–TBG–*Nrf2*/GFP via the tail vein and fed with HFD for 12 weeks. (B) Body weight changes. (C) GTT and AUC_glucose_. (D) ITT and AUC_insulin_. (E) Hepatic H&E and Oil‐red‐O staining. CV, central vein. (F) IF staining for F4/80 in the liver. (G) Hepatic mRNA expression for pyroptotic cell markers. (H) Western blots analysis for pyroptotic cell markers in the liver. (I) Co‐IF for GSDMD with ALB in the liver. (J) IF staining for IL1β in the liver. HFD, high‐fat diet; GTT, glucose tolerance test; ITT, insulin tolerance test; AUC, areas under the glucose curves. Data are expressed as the mean ± SEM (n = at least 3 mice and 3 independent experiments). ns, not significant. *p < .05, **p < .01, ***p < .001.

To further determine the impact of *Nrf2* overexpression on pyroptosis in the liver, we generated liver specific *Nrf2* overexpression mice. Eight‐week‐old male WT mice were injected via the tail vein with adeno‐associated virus carrying the TBG promotor and expressing *Nrf2*/GFP (AAV8–TBG–*Nrf2*/GFP) referred as AAV–*Nrf2*. These mice were then fed a HFD for 12 weeks (Figure 4A). The efficiency of *Nrf2* overexpression was confirmed (Figure S4A–C). AAV–*Nrf2* mice exhibited reduced body weight gain (Figure 4B) and improved glucose tolerance and clearance, as evidenced by GTT and ITT (Figure 4C,D). Furthermore, *Nrf2* overexpression in the liver mitigated HFD‐induced liver steatosis and macrophage infiltration, as shown by histological analyses (Figure 4E,F). Additionally, it reduced the tendency towards hepatic fibrosis, as indicated by Sirius red and α‐SMA staining (Figure S4D). Overexpression of *Nrf2* also resulted in a significant down‐regulation of genes related to gluconeogenesis and fat formation, while glycolysis‐related genes remained unchanged (Figure S4E–G). These results indicate that hepatic *Nrf2* overexpression enhances lipid degeneration and insulin sensitivity in the context of HFD.

To further investigate the effects of *Nrf2* overexpression on hepatic pyroptosis, we determined the expression of pyroptosis marker genes. Overexpression of *Nrf2* resulted in reduced mRNA levels of *Gsdmd*, *Il18* and *Il1β*, as well as reduced protein levels of cle‐Caspase1, GSDMD, N‐GSDMD, Pro‐IL1β and IL1β protein expression in the liver (Figures 4G,H and S4H). The decreased cle‐Caspase1, GSDMD and IL1β were confirmed by IF (Figures 4I,J and S4I). Moreover, TUNEL staining showed decreased cell death in the liver (Figure S4J). These findings demonstrate that NRF2 overexpression mitigates HFD‐induced hepatocyte pyroptosis and inflammation.

### 3.5 NRF2 regulates hepatocyte pyroptosis in vitro

**FIGURE 5:**
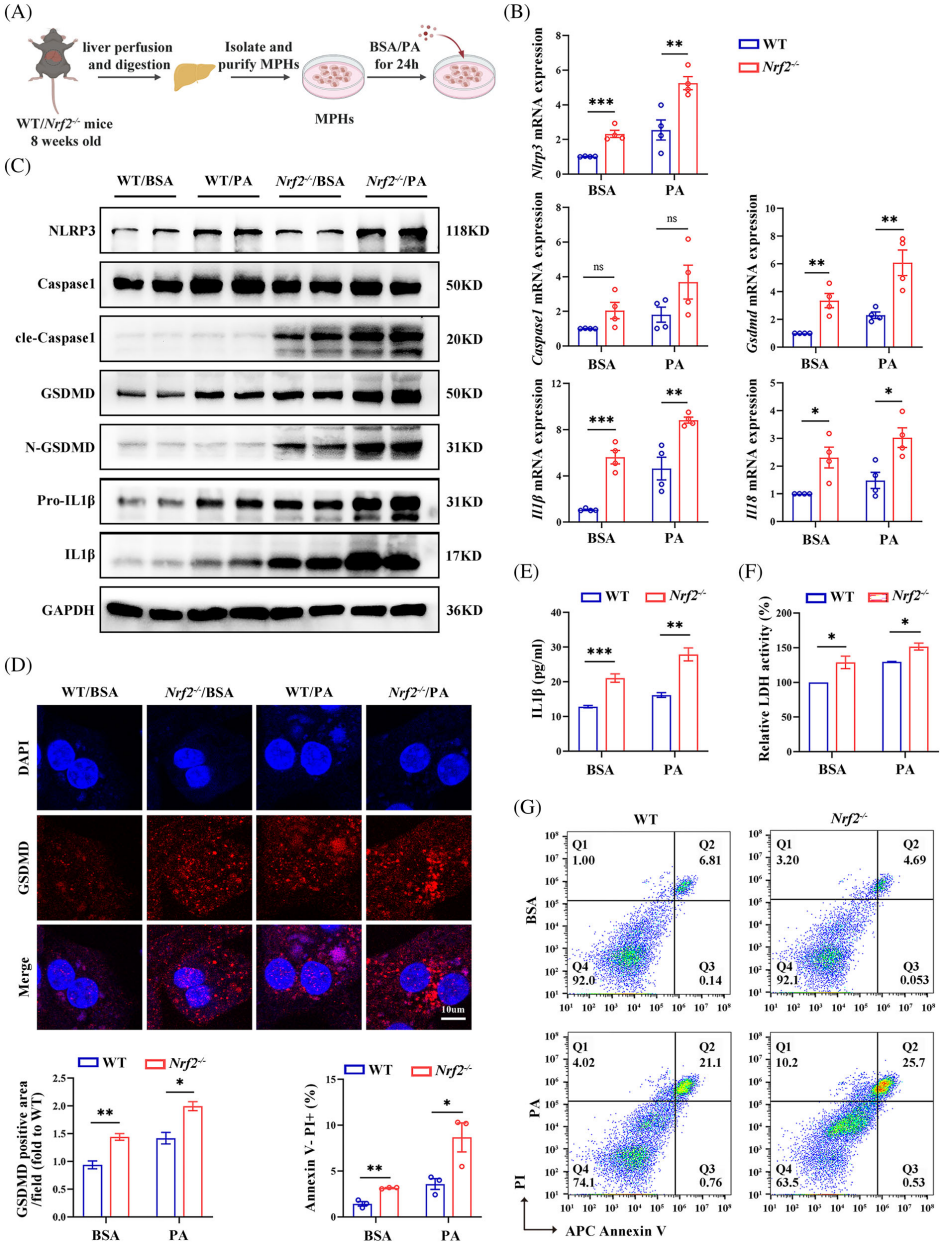
MPHs from *Nrf2^−/−^* mice exhibit increased pyroptosis and inflammation. (A) Schematic diagram of MPHs isolation and treatments. MPHs were isolated from WT or *Nrf2^−/−^* mice and then treated with PA or BSA for 24 h. (B) mRNA expression for pyroptosis markers. (C) Western blots analysis for pyroptosis markers. (D) IF staining for GSDMD. (E) IL1β protein levels in culture medium. (F) LDH activity in the culture medium. (G) Flow cytometry for Annexin V/PI‐positive cells. MPHs, mouse primary hepatocytes; LDH, lactate dehydrogenase. PA, palmitic acids; data are expressed as the mean ± SEM (*n* = 3 mice and three independent experiments). ns, no significant. *p < .05, **p < .01, ***p < .001.

To confirm role of *Nrf2* in regulating pyroptosis in vitro, we isolated MPHs from *Nrf2^−/−^* and WT mice and treated them with or without PA to induced pyroptosis (Figure 5A). To confirm the appropriate concentration of PA, we conducted a CCK8 cell viability assay. The results showed that in MPHs, the cell survival rate significantly decreased when the PA concentration exceeded 200 µM. Therefore, MPHs were treated with 200 µM PA (Figure S5A). Furthermore, We found that with the increase of PA concentration, the expression of pyroptosis marker protein also increased (Figure S5B). As expected, NRF2 expression was almost invisible in *Nrf2^−/−^* MPHs (Figure S5C,D). Consistence with in vivo findings, *Nrf2* deficiency enhanced PA‐induced mRNA expressions of genes related to gluconeogenesis, while glycolysis‐related genes remained unchanged (Figure S5E,F). Additionally, the mRNA expression of genes related to lipid metabolism and intracellular lipid droplets significantly increased (Figure S5G,H). Prompted by in vivo results, we further determine whether NRF2 regulates pyroptosis at the cellular level. MPHs from WT or *Nrf2^−/−^* mice were used for this analysis. Notably, MPHs from *Nrf2^−/−^* mice exhibited elevated mRNA expression of *Nlrp3*, *Gsdmd*, *Il18* and *Il1β* under both BSA and PA treatment compared with MPHs from WT mice (Figure 5B). Furthermore, PA‐treated MPHs from *Nrf2^−/−^* mice showed increased protein levels of NLRP3, cle‐Caspase1, GSDMD, N‐GSDMD, pro‐IL1β and IL1β (Figures 5C and S5I). IF staining confirmed increased GSDMD expression in *Nrf2^−/−^* MPHs (Figure 5D). Moreover, supernatants from the PA‐treated MPHs of *Nrf2^−/−^* mice showed increased IL1β expression and LDH activity (Figure 5E,F). Finally, an increased proportion of Annexin V^−^/PI^+^ cell, indicative of pyroptosis,^21^ was observed in MPHs from *Nrf2^−/−^* mice (Figure 5G). These in vitro results suggest that loss of *Nrf2* promotes glucose/lipid metabolism disorders, inflammatory responses and pyroptosis, especially under high‐fat conditions.

**FIGURE 6:**
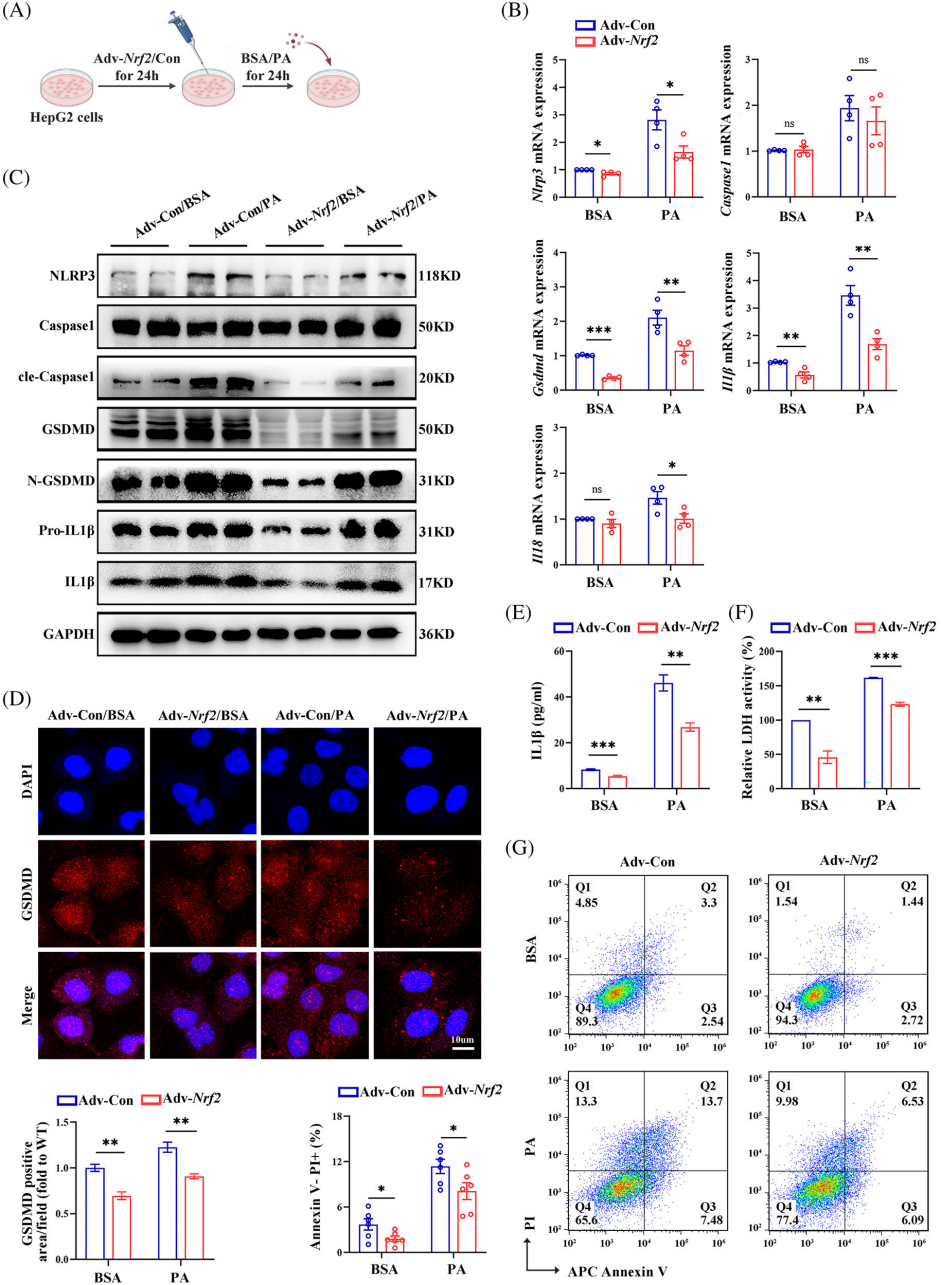
*Nrf2* overexpression inhibits pyroptosis and inflammation in HepG2 cells. (A) Schematic diagram of the study procedure. HepG2 cells were transfected with adenovirus encoding a control vector (Adv‐Con) or *Nrf2* (Adv‐*Nrf2*) for 48 h following by administration of BSA or PA for 24 h. (B) mRNA expression for pyroptosis markers. (C) Western blots analysis for pyroptosis markers. (D) IF staining for GSDMD. (E) IL1β protein levels in culture medium. (F) LDH activity in the culture medium. (G) Flow cytometry for AnnexinV/PI‐positive cells. Con, controls; LDH, lactate dehydrogenase. PA, palmitic acids; BSA, bovine serum albumin. Data are expressed as the mean ± SEM (*n* = 3 independent experiments). ns, not significant. *p < .05, **p < .01, ***p < .001.

To further explore the role of NRF2 in different cell types, HepG2 cells were transfected with Adv expressing *Nrf2*/Control (Adv–*Nrf2/*Con) and treated with PA/BSA for 24 h (Figure 6A). In addition, the CCK8 experiments showed that a PA concentration of 400 µM significantly reduced the survival rate of HepG2 cells (Figure S6A). With the increase of PA concentration, the expression of pyroptosis marker proteins and lipid deposition increased (Figure S6B,C). The efficiency of transfection was shown in Figure S6D–F. In PA‐treated HepG2 cells, *Nrf2* overexpression significantly down‐regulated genes related to gluconeogenesis and fat synthesis, reduced intracellular lipid droplets, while glycolysis‐related genes remained comparable between groups (Figure S6G–J). Furthermore, *Nrf2* overexpression in HepG2 cells also led to down‐regulation of PA‐induced pyroptosis markers and inflammatory cytokine expression (Figures 6A–G and S6K). Together, these data suggest that NRF2 impacts glucose/lipid metabolism at least partially through its effect on pyroptosis in the liver.

### 3.6 NRF2 regulates GSDMD expression at the transcription level

**FIGURE 7:**
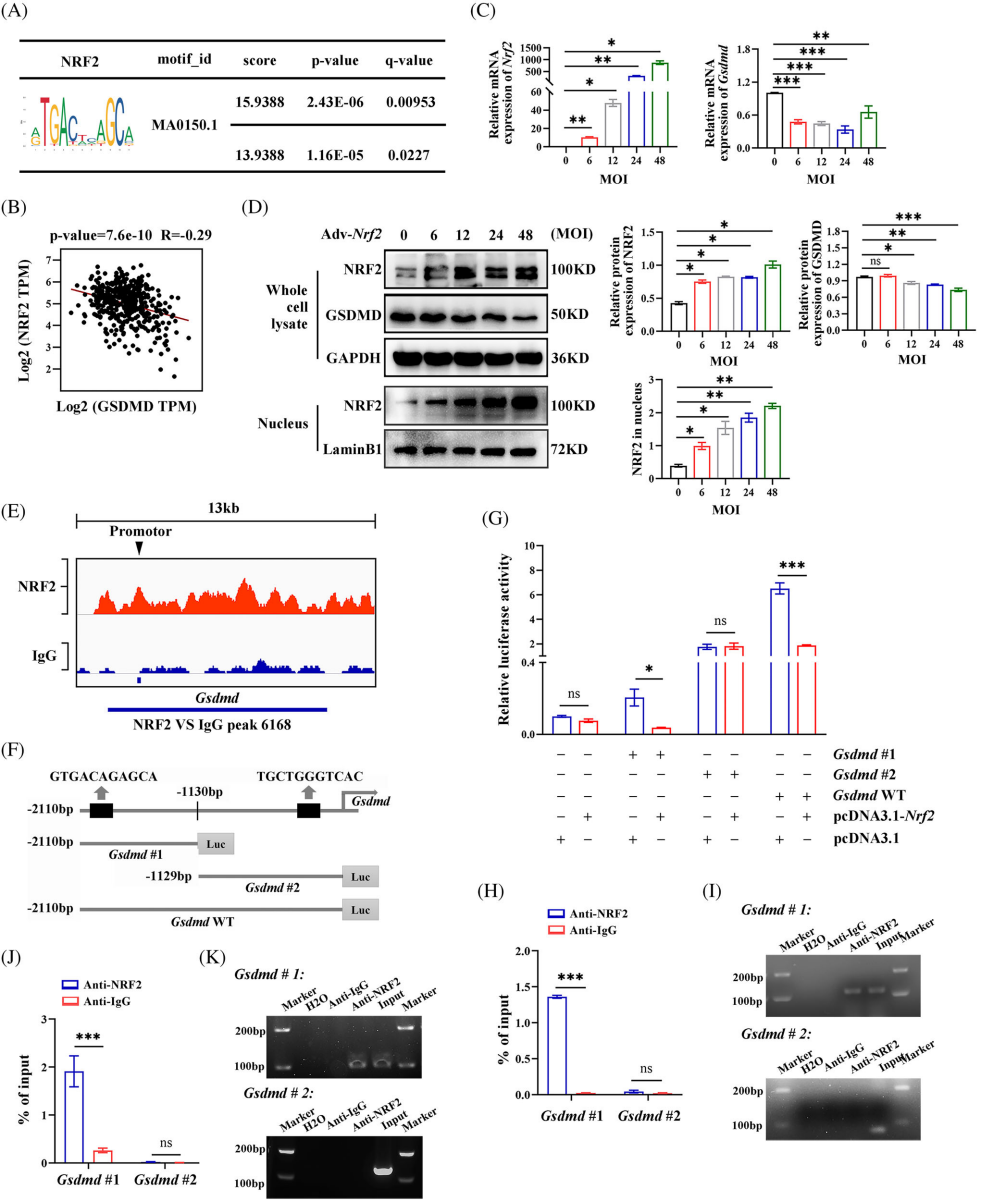
NRF2 inhibits transcription and expression of GSDMD in vitro. (A) Prediction of NRF2's binding motifs and the promoters of pyroptosis‐related genes in JASPAR database (https://jaspar.elixir.no/). (B) Prediction of the relationship between *Nrf2* and *Gsdmd* mRNA expression in GEPIA database (http://gepia.cancer‐pku.cn/). (C and D) HepG2 cells were transfected with Adv‐*Nrf2* at increasing MOI (6, 12, 24, 48) for 48 h, as indicated in the Methods. NRF2 (both in whole cell lysate and nucleus) and GSDMD mRNA (C) and protein (D) expression levels. (E) ChIP‐seq analysis of the binding of NRF2 on the promoters of *Gsdmd* in HepG2 cells. (F) Schematic diagram showing luciferase reporter vectors containing truncated (*Gsdmd*#1, −2110 to −1030 bp; *Gsdmd*#2, −1129 to 0 bp) or *Gsdmd* WT promoters. (G) The relative luciferase activities in HepG2 cells co‐transfected with luciferase reporter plasmids containing WT or truncated *Gsdmd* promoter sequences and overexpression plasmids of *Nrf2*. (H) ChIP‐qPCR assay for the binding of NRF2 to *Gsdmd* promoter in HepG2 cells. (I) Agarose gel electrophoresis for detecting NRF2's binding to *Gsdmd* promoter with primers for *Gsdmd*#1 and *Gsdmd*#2. (J) ChIP‐qPCR assay for the binding of NRF2 to *Gsdmd* promoter in the liver of WT mice transfected with AAV–TBG–*Nrf2*. (K) Agarose gel electrophoresis for detecting NRF2's binding to *Gsdmd* promoter with primers for *Gsdmd*#1 and *Gsdmd*#2 in liver of WT mice. MOI, multiplicity of infection. Data are expressed as the mean ± SEM (n = 3 independent experiments). ns, not significant. *p < .05, **p < .01, ***p < .001.

The previous findings revealed that NRF2 inhibits the expression of pyroptosis marker genes. To understand the underlying mechanism, we investigated whether NRF2 transcriptionally regulates these genes. Using the JASPAR database, we predicted potential NRF2 binding sites within the promoters of pyroptosis‐related genes (Table S4). Notably, the *Gsdmd* promoter showed the highest relative profile score for the NRF2 binding motif (ID: MA0150.1), indicating that NRF2 may bind to the *Gsdmd* promoter (Figure 7A). In addition, a negative correlation between *Nrf2* and *Gsdmd* mRNA expression was found in the GEPIA database (http://gepia.cancer‐pku.cn/) (Figure 7B). Further experiments in HepG2 cells showed that with the Adv‐*Nrf2* infection titer increases, the expression of GSDMD mRNA and protein decreases. (Figure 7C,D). These findings, consistent with our in vivo and in vitro results, indicate that NRF2 negatively regulates GSDMD expression at the transcriptional level. To confirm that NRF2 can bind to the *Gsdmd* promoter, we performed a ChIP‐seq analysis using an anti‐NRF2 antibody in HepG2 cells, revealing a peak at the *Gsdmd* promoter locus (Figure 7E). To identify specific NRF2 binding sites within the *Gsdmd* promoter, we conducted luciferase reporter assays. We constructed luciferase reporter vectors containing the full‐length *Gsdmd* promoter (WT), a −2110 to −1130 bp fragment (*Gsdmd*#1) and −1129 to 0 bp fragment (*Gsdmd*#2) (Figure 7F). The luciferase assays showed that overexpression of *Nrf2* significantly inhibited the transcriptional activity of *Gsdmd*#1 and the *Gsdmd* WT but did not affect the activity of *Gsdmd*#2 (Figure 7G). To further verify NRF2 binding to the *Gsdmd* promotor, we performed ChIP‐qPCR assay in HepG2 cells using an NRF2 antibody. As expected, NRF2 primarily bound to the *Gsdmd*#1 fragment of the promotor (Figure 7H,I). ChIP‐qPCR assay was also performed in the liver of AAV–TBG–*Nrf2‐*transfected WT mice to confirm the binding of NRF2 and *Gsdmd*#1 fragment (Figure 7J,K). These data indicate that NRF2 inhibits GSDMD expression by binding to the *Gsdmd*#1 (−2110 to −1130 bp) region of its promoter.

### 3.7 GSDMD‐mediated NRF2's role in regulating hepatocyte pyroptosis

**FIGURE 8:**
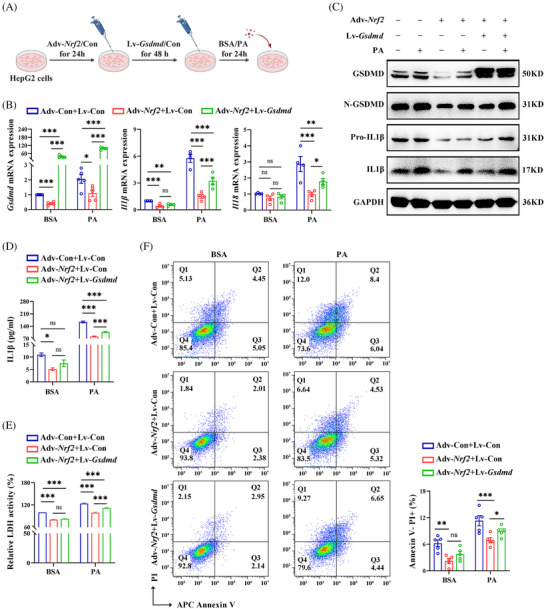
*Gsdmd* overexpression abrogates the inhibitory effects of NRF2 on pyroptosis and inflammation in HepG2 cells. (A) HepG2 cells were transfected with Adv‐*Nrf2*/Con or/and Lv‐*Gsdmd*/Con, as indicated in the Methods. (B) The mRNA expression for pyroptosis markers and inflammatory factor. (C) Western blots analysis for GSDMD, N‐GSDMD, Pro‐IL1β and IL1β. (D) IL1β protein levels in culture medium. (E) LDH activity in the culture medium. (F) Flow cytometry for AnnexinV/PI—positive cells. Con, controls. Data are expressed as the mean ± SEM (*n* = at least 3 independent experiments). ns, no significant. *p < .05, **p < .01, ***p < .001.

To explore whether GSDMD is involved in NRF2‐regulated pyroptosis, we transfected HepG2 cells overexpressing *Nrf2* with lentivirus expressing *Gsdmd* (Lv‐*Gsdmd*) (Figure 8A). The efficiency of transfection was confirmed (Figure S7A,B). Our results revealed that *Gsdmd* overexpression significantly counteracted the effect of NRF2 on liver lipid deposition (Figure S7C). In addition, *Gsdmd* overexpression abrogated the inhibition effect of NRF2 on the mRNA expression of inflammatory factors, as well as on the protein levels of Pro‐IL1β and IL1β (Figures 8B,C and S7D). Furthermore, *Gsdmd* overexpression nullified the effects of Adv‐*Nrf2* treatment on IL1β levels, LDH activity in cell medium and the proportion of Annexin V^−^/PI^+^ cells in responding to PA (Figure 8D–F). These observations highlight NRF2's role in the regulation of lipid metabolism, chronic inflammation and pyroptosis are GSDMD‐dependent in HepG2 cells.

**FIGURE 9:**
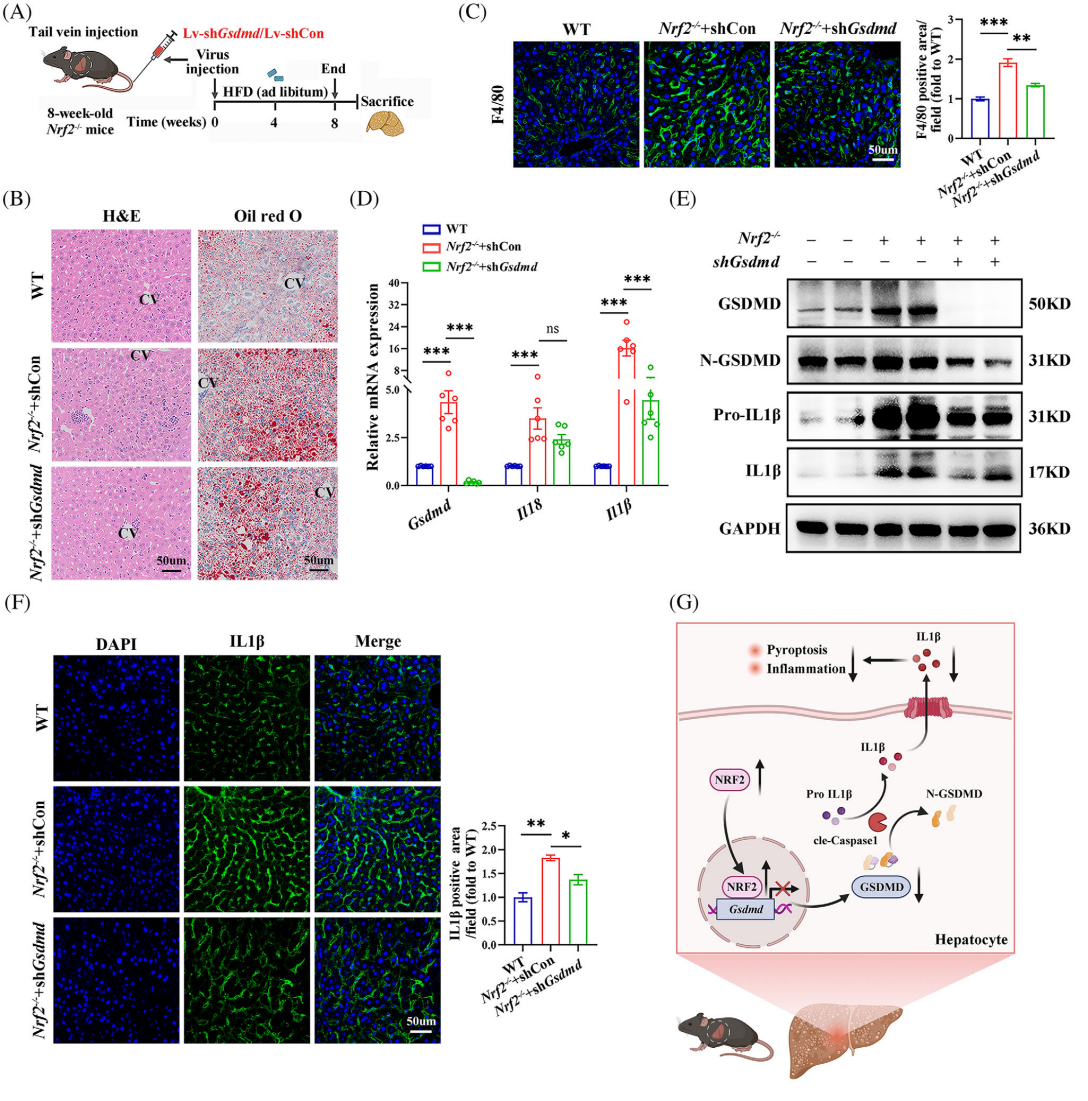
*Gsdmd* knockdown mitigates the increased pyroptosis in *Nrf2^−/−^* mice. (A) Schematic diagram of the study procedure. Eight‐week‐old male WT and *Nrf2^−/−^* mice were injected with LV‐sh*Gsdmd*/shCon via the tail vein and fed with HFD for −8 weeks. (B) H&E and Oil‐red‐O staining in the liver. CV, central vein. (C) IF staining for F4/80 in the liver. (D) Hepatic mRNA expression for *Gsdmd, Il18 and Il1β*. (E) Western blots analysis for GSDMD, N‐GSDMD, Pro‐IL1β and IL1β in the liver. (F) IF staining for IL1β in the liver. (G) Schematic model; NRF2 binds to the *Gsdmd* promoter, inhibiting the GSDMD expression and thereby improving glucose/lipid metabolism and liver steatosis. Con, control; HFD, high‐fat diet. Data are expressed as the mean ± SEM (*n* = 3 mice or three independent experiments). *p < .05, **p < .01, ***p < .001.

To examine the influence of GSDMD on NRF2‐related pyroptosis in vivo, 8‐week‐old male *Nrf2^−/−^* mice were administered Lv‐*shGsdmd/*shCon via tail vein injection and maintained on a HFD for 8 weeks (Figure 9A). Transfection efficiency was shown in Figure S8A–C. Down‐regulation of *Gsdmd* significantly mitigated *Nrf2* deficiency‐induced hepatic steatosis and macrophage infiltration in the liver of HFD‐fed mice (Figures 9B,C and S8D). Additionally, *Gsdmd* knockdown alleviated the up‐regulation of *Il1β* mRNA expression, as well as the protein levels of Pro‐IL1β, and IL1β, which were elevated due to *Nrf2* deficiency in the liver of these animals (Figures 9D,E and S8E). Confocal imaging confirmed that *Gsdmd* knockdown reversed the effects of *Nrf2* deficiency on IL1β (Figure 9F). Overall, these results demonstrate that NRF2 regulates hepatocyte pyroptosis and associated inflammation in a GSDMD‐dependent manner (Figure 9G).

## 4 DISCUSSION

Pyroptosis is a newly identified form of programmed cell death.^7^ While chronic low‐grade inflammation and hepatic steatosis may trigger pyroptosis, its precise mechanism remain unclear. NRF2 is known to have protective effects against hepatic steatosis, largely by inhibiting chronic inflammation.^22^ However, it is currently unknown whether NRF2 exerts its effect by inhibiting pyroptosis in the liver. In this study, we demonstrate that NRF2 plays a regulatory role in liver steatosis, chronic low‐grade inflammation and IR. Our finding suggests that NRF2 regulates liver glucose and lipid metabolism through a novel mechanism involving pyroptosis. Mechanistically, we found that NRF2 binds to the *Gsdmd* promoter fragment (−2110 to −1130 bp), thereby inhibiting its transcription, leading to reduced pyroptosis and lipid deposition in the liver. These findings broaden our understanding of the pathological role of NRF2 in MAFLD.

Previous studies have highlighted the pivotal role of NRF2 in the progression of MAFLD and MASH with fibrosis.^23^, ^24^ NRF2 is primarily known for its involvement in oxidant responses, where it suppresses oxidative stress by transcriptionally up‐regulating antioxidant enzymes, such as HO‐1, NADPH and GSH9^9^. Additionally, NRF2 activation reduces inflammation by either decreasing oxidative responses or directly inhibiting inflammatory pathways, such as the NFκB signaling pathway.^25^ These mechanisms contribute to increased insulin sensitivity and enhanced metabolism of glucose and lipids. However, evidence regarding the role of NRF2 in regulating these process is contradictory. Some studies, including our previous research, indicated that NRF2 can improve liver steatosis, inhibit fat production and liver fibrosis and exert anti‐inflammatory and antioxidant effects.^15^, ^22^, ^26^ Several NRF2 activators, such as TBE‐31 and CDDOIm, have been reported to alleviate HFD‐induced MAFLD by reducing liver steatosis, inflammation, lipid deposition and fibrosis.^9^, ^27^ Conversely, other studies report that NRF2 has minimal or even adverse effects on glucose and lipid metabolism and diet‐induced obesity.^26^, ^28^ In this study, we confirmed the positive regulatory role of NRF2 in lipid metabolism using both gain‐ and loss‐of‐function approaches in vitro and in vivo. Importantly, EHC experiments revealed that *Nrf2* deficiency increases HGP and IR under HFD feeding, supporting the beneficial effects of NRF2 on glucose metabolism and IR in diet‐induced obesity.

Pyroptosis and inflammasome activation have recently been recognised as significant porcesses in liver disease such as MAFLD and MASH.^29^ Pyroptosis is marked by membrane disruption, caspase activation, pore formation, cellular swelling and the secretion of pro‐inflammatory cytokines.^30^, ^31^, ^32^ Remarkably, the suppression of pyroptosis effectors, with inhibitors like MCC950 for NLRP3, Ac‐YVAD‐cmk for Caspase‐1 and necrosulfonamide for GSDMD, has shown to be protective against MAFLD.^33^ Recent studies have linked pyroptosis to MAFLD progression, associating lipid deposition with increased inflammation and fibrosis.^34^ This might be largely related to the increased inflammation level caused by pyroptosis, which contributes to IR in MAFLD.^35^

Similar to previous reports, our study found significant changes in inflammation and pyroptosis markers in the liver of MAFLD patients and obese mice, suggesting increased pyroptosis accompanied by chronic inflammation in liver steatosis. NRF2 expression also increased the liver of these mice, especially in hepatocytes, suggesting a link between NRF2 and pyroptosis in the liver. As expected, overexpression of NRF2 improved liver steatosis in diet‐induced obese mice, while inhibiting pyroptosis and inflammation markers, including cle‐Caspase1, GSDMD, N‐GSDMD, Pro‐IL1β and IL1β. Conversely, liver specific *Nrf2* deficiency showed opposite results. These results demonstrate that the improvement of liver steatosis and glucose/lipid metabolism by NRF2 in obese mice is related to its inhibitory effects on pyroptosis and chronic inflammation. The observed reduction in cleaved Caspase‐1 may be secondary to the overall decrease in oxidative stress and inflammation mediated by NRF2, which showed opposite results in *Nrf2*‐deficient models. Our previous study revealed that *Nrf2* deficiency promotes liver steatosis by enhancing SREBP‐1c activity and reducing autophagy.^15^ Therefore, our findings provided evidence that NRF2 influences liver glucose and lipid metabolism through multiple pathways.

GSDMD, a substrate of inflammatory caspases, is crucial in inflammatory caspase‐mediated pyroptosis^36,37^and the pathogenesis of MAFLD by regulating cytokine secretion and fat generation.^4^ Our study reveals that increased NRF2 expression significantly inhibits GSDMD expression in vitro, indicating that GSDMD expression is regulated by NRF2.

To clarify the relationship between NRF2 and pyroptosis‐related genes, we predicted NRF2 binding to the promoters of these genes using the JASPAR database and found that NRF2 may bind to the *Gsdmd* promoter. We further performed ChIP‐qPCR assay to ascertain the NRF2's binding with *Gsdmd* promoter. Finally, we confirmed that NRF2 inhibits GSDMD expression by binding to the *Gsdmd* (−2110 to −1130 bp) fragment of its promoter. Additionally, our results indicate that the regulation of NRF2 on liver pyroptosis and related inflammation is GSDMD dependent. It has been reported that GSDMD exerts its pyroptosis actuator function by releasing the N‐GSDMD which induces pyroptosis and controls the release of inflammatory factors.^38^, ^39^ Therefore, NRF2 improves liver glucose metabolism and lipogenesis, at least in part, by inhibiting GSDMD expression, reducing N‐GSDMD and pro‐inflammatory cytokine secretion. The NRF2–GSDMD–pyroptosis pathway may thus be key pathway in the pathological mechanism of MAFLD. Based on our previous and other findings,^15^, ^40^, ^41^ it has shown that NRF2 has multiple roles and mechanisms in regulating lipid metabolism and MAFLD. This highlights the complexity of NRF2 regulatory mechanisms. In this study, we demonstrated how NRF2 inhibits HFD‐induced hepatocyte pyroptosis by binding to *Gsdmd* promoter and suppressing GSDMD expression. Therefore, our results provide a new insight into the mechanism by which NRF2 regulates metabolic disorders, contributing to a better understanding of the multifaceted functions of NRF2 in MAFLD.

In summary, our study revealed a new role for NRF2 in inhibiting hepatic glucose production and lipogenesis by inhibiting pyroptosis in the liver. These findings provide insights into the mechanism by which NRF2 regulates liver glucose/lipid metabolism. In addition, developing drugs targeting NRF2 may offer an effective multi‐target approach for treating MAFLD, MASH and obesity.

This study has some limitations: (1) We have not performed in‐depth study on the molecular mechanisms of glucose metabolism in the liver, necessitating further investigation; (2) due to the small sample size in human liver tissue, we did not analyse the correlation between NRF2 expression and other clinical features. Therefore, further clinical cohort studies are needed. Finally, before NRF2 is considered as a potential pathway for clinical treatment of MAFLD and obesity, its protective effects need to be further evaluated in large animal studies.

## AUTHOR CONTRIBUTIONS

X. X. performed the experiments and analysed the data. Q. Z. and X. F. performed the experiments and analysed the data. G. Y. and L. L. contributed to data acquisition and analysis and revised the manuscript. X. X. and M. Y. contributed to conception, data interpretation and revised the manuscript.

## CONFLICT OF INTEREST STATEMENT

The authors declared no conflicts of interest.

## Supporting information



Supporting Information

Supporting Information

Supporting Information

Supporting Information

Supporting Information

Supporting Information

Supporting Information

Supporting Information

Supporting Information

## Data Availability

The data that support the findings of this study are presented in the main text and supporting information. All the original data are available from the corresponding author upon reasonable request.

## References

[1] Schuster S , Cabrera D , Arrese M , Feldstein AE . Triggering and resolution of inflammation in NASH. Nat Rev Gastroenterol Hepatol. 2018;15(6):349‐364. 29740166 10.1038/s41575-018-0009-6

[2] Eguchi A , Wree A , Feldstein AE . Biomarkers of liver cell death. J Hepatol. 2014;60(5):1063‐1074. 24412608 10.1016/j.jhep.2013.12.026

[3] Yang W , Liu L , Wei Y , et al. Exercise suppresses NLRP3 inflammasome activation in mice with diet‐induced NASH: a plausible role of adropin. Lab Invest. 2021;101(3):369‐380. 33268842 10.1038/s41374-020-00508-y

[4] Xu B , Jiang M , Chu Y , et al. Gasdermin D plays a key role as a pyroptosis executor of non‐alcoholic steatohepatitis in humans and mice. J Hepatol. 2018;68(4):773‐782. 29273476 10.1016/j.jhep.2017.11.040

[5] Beier JI , Banales JM . Pyroptosis: An inflammatory link between NAFLD and NASH with potential therapeutic implications. J Hepatol. 2018;68(4):643‐645. 29408544 10.1016/j.jhep.2018.01.017 PMC6185810

[6] Aziz M , Jacob A , Yang WL , Matsuda A , Wang P . Current trends in inflammatory and immunomodulatory mediators in sepsis. J Leukoc Biol. 2013;93(3):329‐342. 23136259 10.1189/jlb.0912437 PMC3579020

[7] Li R , Xue W , Wei H , et al. Research progress of pyroptosis in fatty liver disease. Int J Mol Sci. 2023;24(17). 10.3390/ijms241713065 PMC10488074 37685870

[8] Knorr J , Wree A , Feldstein AE . Pyroptosis in steatohepatitis and liver diseases. J Mol Biol. 2022;434(4):167271. 34592216 10.1016/j.jmb.2021.167271

[9] Sharma RS , Harrison DJ , Kisielewski D , et al. Experimental nonalcoholic steatohepatitis and liver fibrosis are ameliorated by pharmacologic activation of Nrf2 (NF‐E2 p45‐related factor 2). Cell Mol Gastroenterol Hepatol. 2018;5(3):367‐398. 29552625 10.1016/j.jcmgh.2017.11.016 PMC5852394

[10] Shi H , Zhang Y , Xing J , et al. Baicalin attenuates hepatic injury in non‐alcoholic steatohepatitis cell model by suppressing inflammasome‐dependent GSDMD‐mediated cell pyroptosis. Int Immunopharmacol. 2020;81:106195. 32028242 10.1016/j.intimp.2020.106195

[11] Chowdhry S , Nazmy MH , Meakin PJ , et al. Loss of Nrf2 markedly exacerbates nonalcoholic steatohepatitis. Free Radic Biol Med. 2010;48(2):357‐371. 19914374 10.1016/j.freeradbiomed.2009.11.007

[12] Teng Y , Li N , Wang Y , et al. NRF2 inhibits cardiomyocyte pyroptosis via regulating CTRP1 in sepsis‐induced myocardial injury. Shock. 2022;57(4):590‐599. 34907120 10.1097/SHK.0000000000001901

[13] Zhang D , Mao F , Wang S , Wu H , Wang S , Liao Y . Role of transcription factor Nrf2 in pyroptosis in spinal cord injury by regulating GSDMD. Neurochem Res. 2023;48(1):172‐187. 36040608 10.1007/s11064-022-03719-5

[14] Hu JJ , Liu X , Xia S , et al. FDA‐approved disulfiram inhibits pyroptosis by blocking gasdermin D pore formation. Nat Immunol. 2020;21(7):736‐745. 32367036 10.1038/s41590-020-0669-6 PMC7316630

[15] Qiu S , Liang Z , Wu Q , et al. Hepatic lipid accumulation induced by a high‐fat diet is regulated by Nrf2 through multiple pathways. FASEB J. 2022;36(5):e22280. 35394671 10.1096/fj.202101456R

[16] He Y , Zhang C , Luo Y , et al. Hypothalamic BMP9 suppresses glucose production by central PI3K/Akt/mTOR pathway. J Endocrinol. 2021;248(2):221‐235. 33337347 10.1530/JOE-19-0591

[17] Lai Y , Zhao A , Tan M , et al. DOCK5 regulates energy balance and hepatic insulin sensitivity by targeting mTORC1 signaling. EMBO Rep. 2020;21(2):e49473. 31885214 10.15252/embr.201949473 PMC7001503

[18] Cathcart B , Cheedipudi SM , Rouhi L , Zhao Z , Gurha P , Marian AJ . DNA double‐stranded breaks, a hallmark of aging, defined at the nucleotide resolution, are increased and associated with transcription in the cardiac myocytes in LMNA‐cardiomyopathy. Cardiovasc Res. 2024. 10.1093/cvr/cvae063 PMC12641529 38577741

[19] Mohs A , Otto T , Schneider KM , et al. Hepatocyte‐specific NRF2 activation controls fibrogenesis and carcinogenesis in steatohepatitis. J Hepatol. 2021;74(3):638‐648. 33342543 10.1016/j.jhep.2020.09.037

[20] Mridha AR , Wree A , Robertson AAB , et al. NLRP3 inflammasome blockade reduces liver inflammation and fibrosis in experimental NASH in mice. J Hepatol. 2017;66(5):1037‐1046. 28167322 10.1016/j.jhep.2017.01.022 PMC6536116

[21] Xi H , Zhang Y , Xu Y , et al. Caspase‐1 inflammasome activation mediates homocysteine‐induced pyrop‐apoptosis in endothelial cells. Circ Res. 2016;118(10):1525‐1539. 27006445 10.1161/CIRCRESAHA.116.308501 PMC4867131

[22] Xu D , Xu M , Jeong S , et al. The role of Nrf2 in liver disease: novel molecular mechanisms and therapeutic approaches. Front Pharmacol. 2018;9:1428. 30670963 10.3389/fphar.2018.01428 PMC6331455

[23] Hayes JD , Dinkova‐Kostova AT . The Nrf2 regulatory network provides an interface between redox and intermediary metabolism. Trends Biochem Sci. 2014;39(4):199‐218. 24647116 10.1016/j.tibs.2014.02.002

[24] Ramos‐Tovar E , Muriel P . Molecular Mechanisms That Link Oxidative Stress, Inflammation, and Fibrosis in the Liver. Antioxidants (Basel). 2020;9(12). 10.3390/antiox9121279 PMC7765317 33333846

[25] Fan C , Ling‐Hu A , Sun D , et al. Nobiletin ameliorates hepatic lipid deposition, oxidative stress, and inflammation by mechanisms that involve the Nrf2/NF‐kappaB axis in nonalcoholic fatty liver disease. J Agric Food Chem. 2023;71(50):20105‐20117. 38073108 10.1021/acs.jafc.3c06498

[26] Li L , Fu J , Liu D , et al. Hepatocyte‐specific Nrf2 deficiency mitigates high‐fat diet‐induced hepatic steatosis: Involvement of reduced PPARgamma expression. Redox Biol. 2020;30:101412. 31901728 10.1016/j.redox.2019.101412 PMC6940621

[27] Uruno A , Furusawa Y , Yagishita Y , et al. The Keap1‐Nrf2 system prevents onset of diabetes mellitus. Mol Cell Biol. 2013;33(15):2996‐3010. 23716596 10.1128/MCB.00225-13 PMC3719683

[28] Zhang YK , Wu KC , Liu J , Klaassen CD . Nrf2 deficiency improves glucose tolerance in mice fed a high‐fat diet. Toxicol Appl Pharmacol. 2012;264(3):305‐314. 23017736 10.1016/j.taap.2012.09.014 PMC3507999

[29] Hurtado‐Navarro L , Angosto‐Bazarra D , Pelegrín P , Baroja‐Mazo A , Cuevas S . NLRP3 inflammasome and pyroptosis in liver pathophysiology: the emerging relevance of Nrf2 inducers. Antioxidants (Basel, Switzerland). 2022;11(5). 10.3390/antiox11050870 PMC9137763 35624734

[30] Fink SL , Cookson BT . Caspase‐1‐dependent pore formation during pyroptosis leads to osmotic lysis of infected host macrophages. Cell Microbiol. 2006;8(11):1812‐1825. 16824040 10.1111/j.1462-5822.2006.00751.x

[31] Jorgensen I , Miao EA . Pyroptotic cell death defends against intracellular pathogens. Immunol Rev. 2015;265(1):130‐142. 25879289 10.1111/imr.12287 PMC4400865

[32] Shi J , Gao W , Shao F . Pyroptosis: gasdermin‐mediated programmed necrotic cell death. Trends Biochem Sci. 2017;42(4):245‐254. 27932073 10.1016/j.tibs.2016.10.004

[33] Yu L , Hong W , Lu S , et al. The NLRP3 inflammasome in non‐alcoholic fatty liver disease and steatohepatitis: therapeutic targets and treatment. Front Pharmacol. 2022;13:780496. 35350750 10.3389/fphar.2022.780496 PMC8957978

[34] Gaul S , Leszczynska A , Alegre F , et al. Hepatocyte pyroptosis and release of inflammasome particles induce stellate cell activation and liver fibrosis. J Hepatol. 2021;74(1):156‐167. 32763266 10.1016/j.jhep.2020.07.041 PMC7749849

[35] Frankowski R , Kobierecki M , Wittczak A , et al. Type 2 diabetes mellitus, non‐alcoholic fatty liver disease, and metabolic repercussions: the vicious cycle and its interplay with inflammation. Int J Mol Sci. 2023;24(11). 10.3390/ijms24119677 PMC10254034 37298632

[36] Kayagaki N , Stowe IB , Lee BL , et al. Caspase‐11 cleaves gasdermin D for non‐canonical inflammasome signalling. Nature. 2015;526(7575):666‐671. 26375259 10.1038/nature15541

[37] Man SM , Kanneganti TD . Gasdermin D: the long‐awaited executioner of pyroptosis. Cell Res. 2015;25(11):1183‐1184. 26482951 10.1038/cr.2015.124 PMC4650426

[38] Ding J , Wang K , Liu W , et al. Pore‐forming activity and structural autoinhibition of the gasdermin family. Nature. 2016;535(7610):111‐116. 27281216 10.1038/nature18590

[39] Shi J , Zhao Y , Wang K , et al. Cleavage of GSDMD by inflammatory caspases determines pyroptotic cell death. Nature. 2015;526(7575):660‐665. 26375003 10.1038/nature15514

[40] Li L , Fu J , Liu D , et al. Hepatocyte‐specific Nrf2 deficiency mitigates high‐fat diet‐induced hepatic steatosis: Involvement of reduced PPARγ expression. Redox Biol. 2020;30:101412. 31901728 10.1016/j.redox.2019.101412 PMC6940621

[41] Chartoumpekis DV , Ziros PG , Psyrogiannis AI , et al. Nrf2 represses FGF21 during long‐term high‐fat diet‐induced obesity in mice. Diabetes. 2011;60(10):2465‐2473. 21852674 10.2337/db11-0112 PMC3178292

