# Supplementary figures and images for: Nuclear factor erythroid 2‐related factor 2 ameliorates disordered glucose and lipid metabolism in liver: Involvement of gasdermin D in regulating pyroptosis
Source: Clin Transl Med. 2025 Feb 24;15(3):e70233. doi: 10.1002/ctm2.70233 (PMC11850759; doi:10.1002/ctm2.70233)

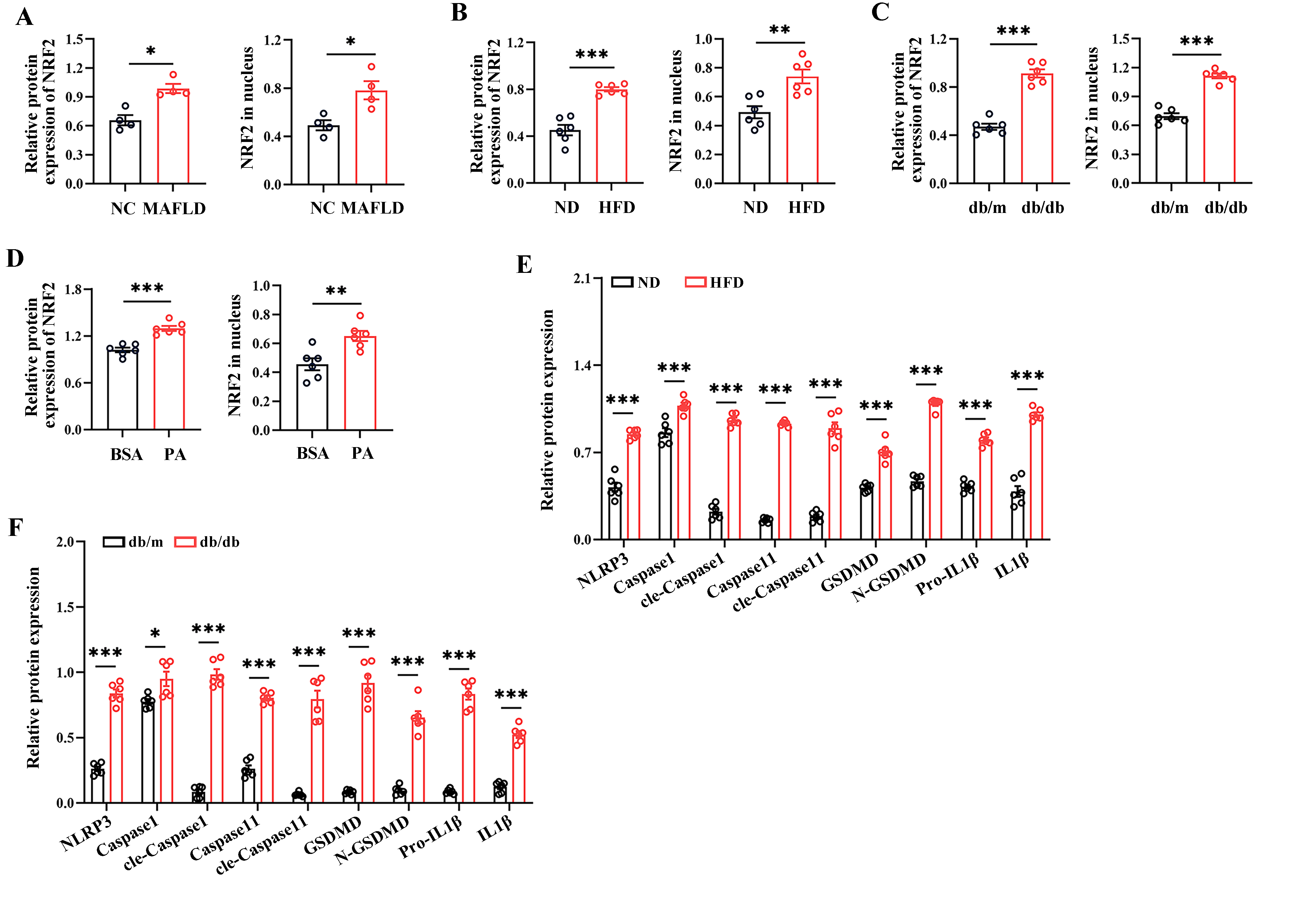

Supplement: Supplementary file 1 — Supporting Information [file CTM2-15-e70233-s004.tiff]

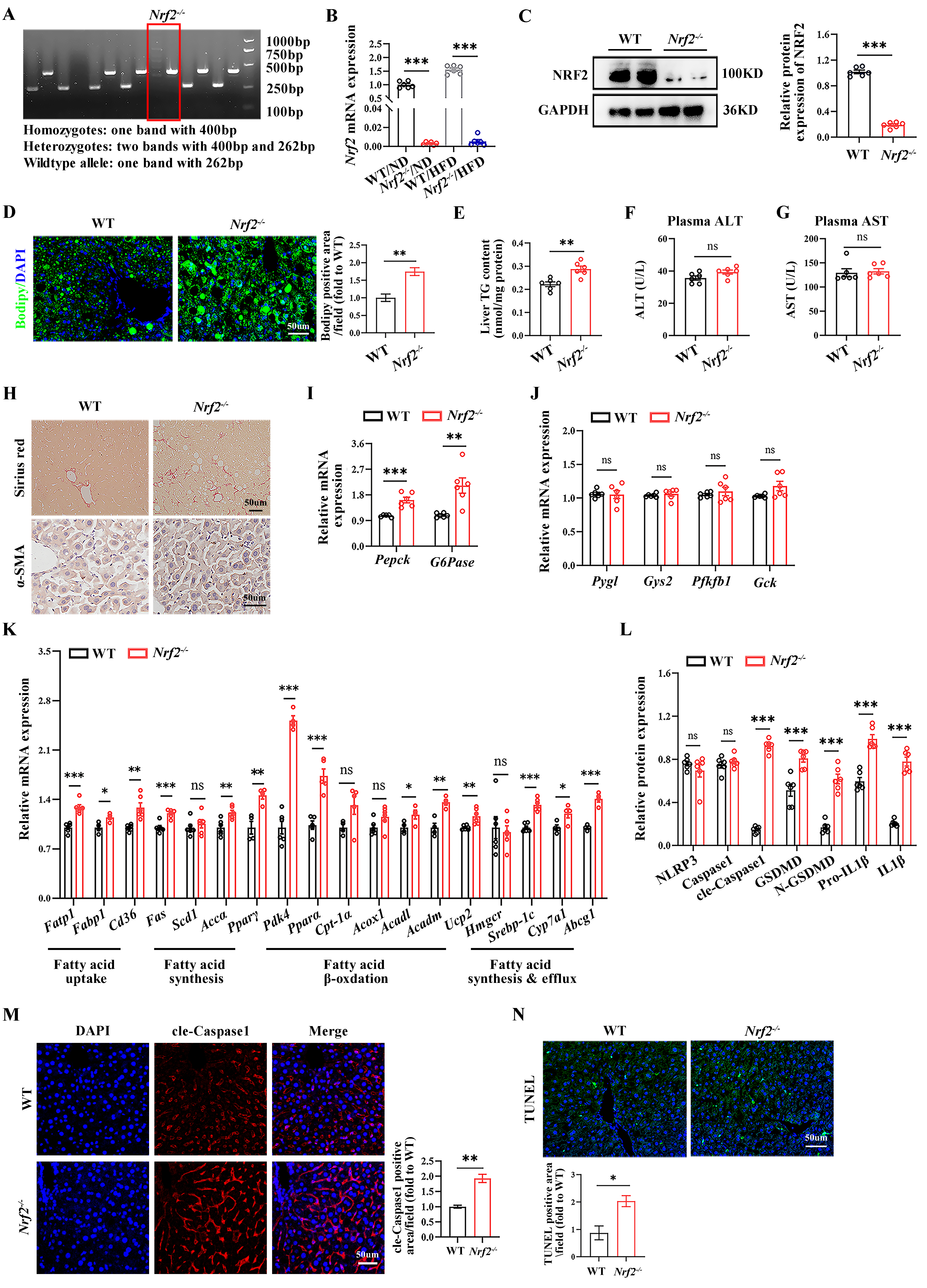

Supplement: Supplementary file 2 — Supporting Information [file CTM2-15-e70233-s009.tif]

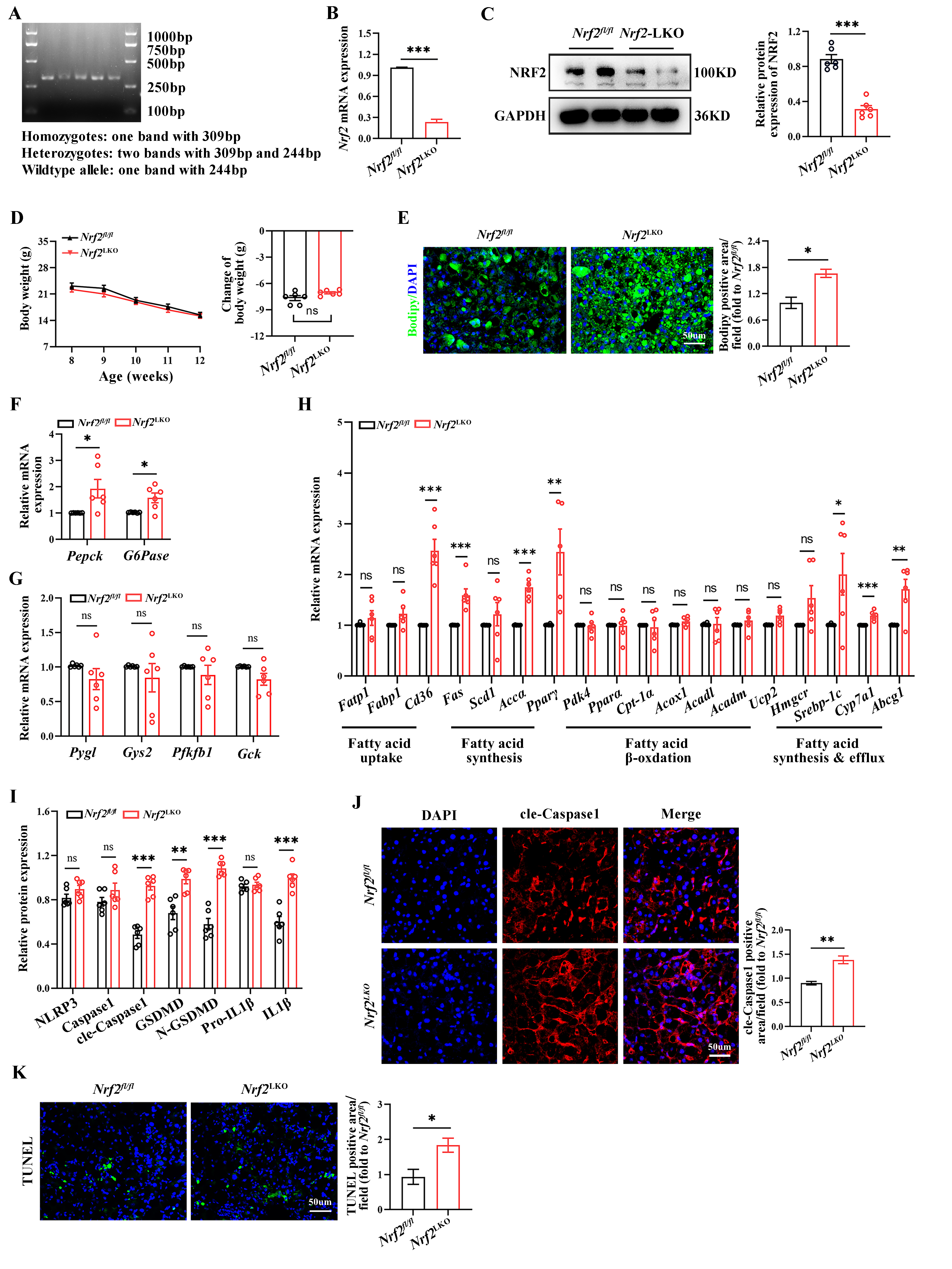

Supplement: Supplementary file 3 — Supporting Information [file CTM2-15-e70233-s005.tif]

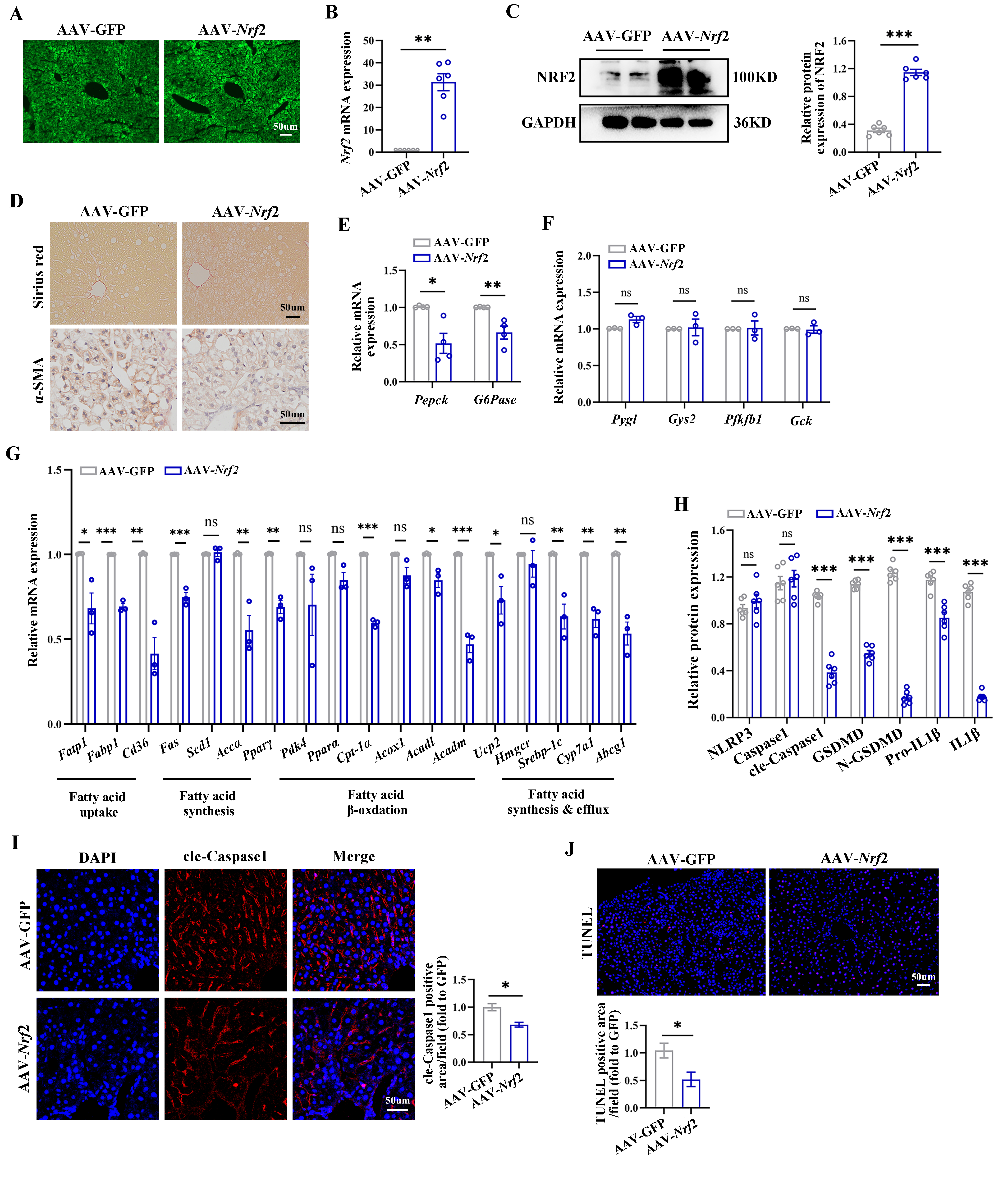

Supplement: Supplementary file 4 — Supporting Information [file CTM2-15-e70233-s006.tiff]

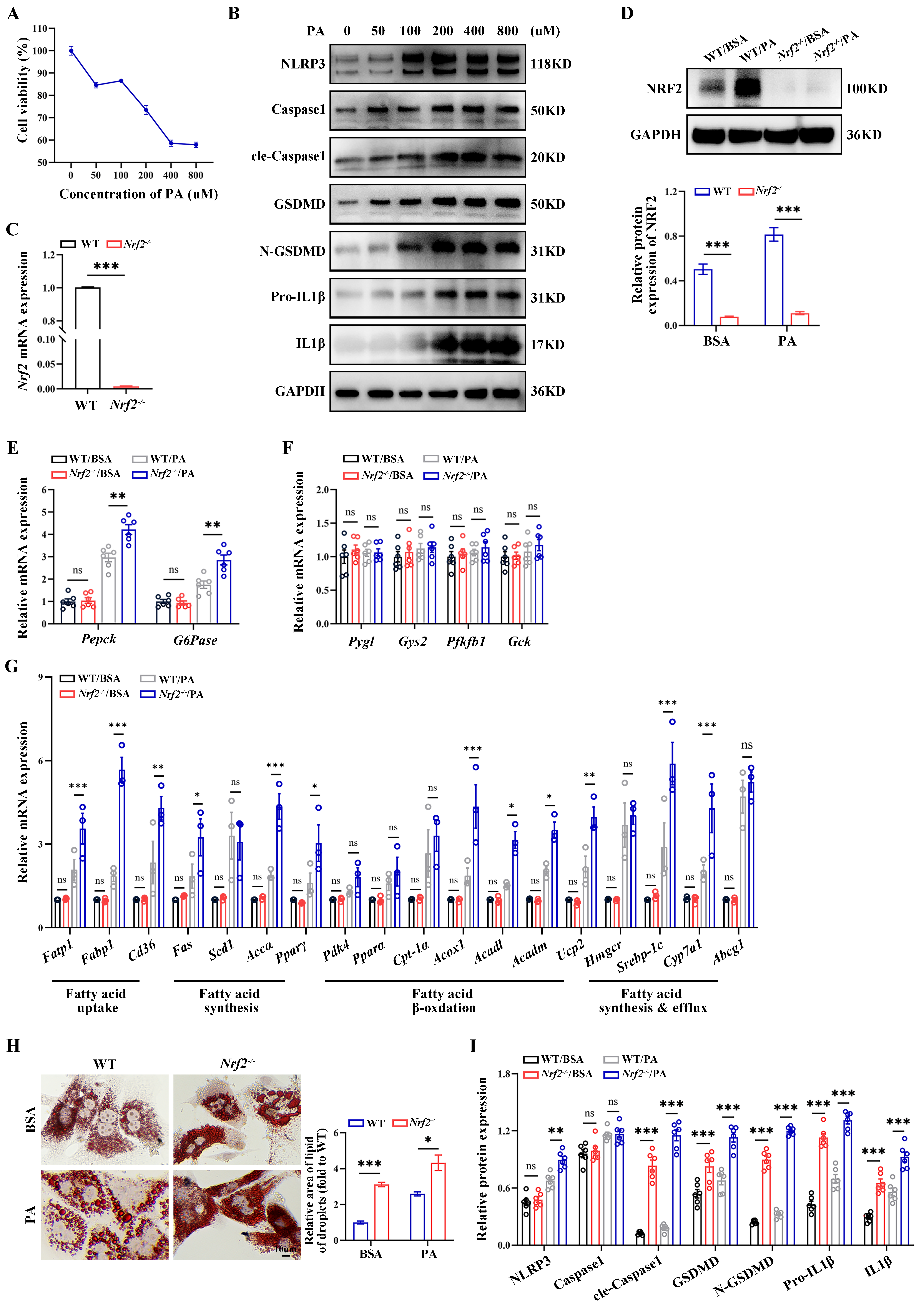

Supplement: Supplementary file 5 — Supporting Information [file CTM2-15-e70233-s003.tif]

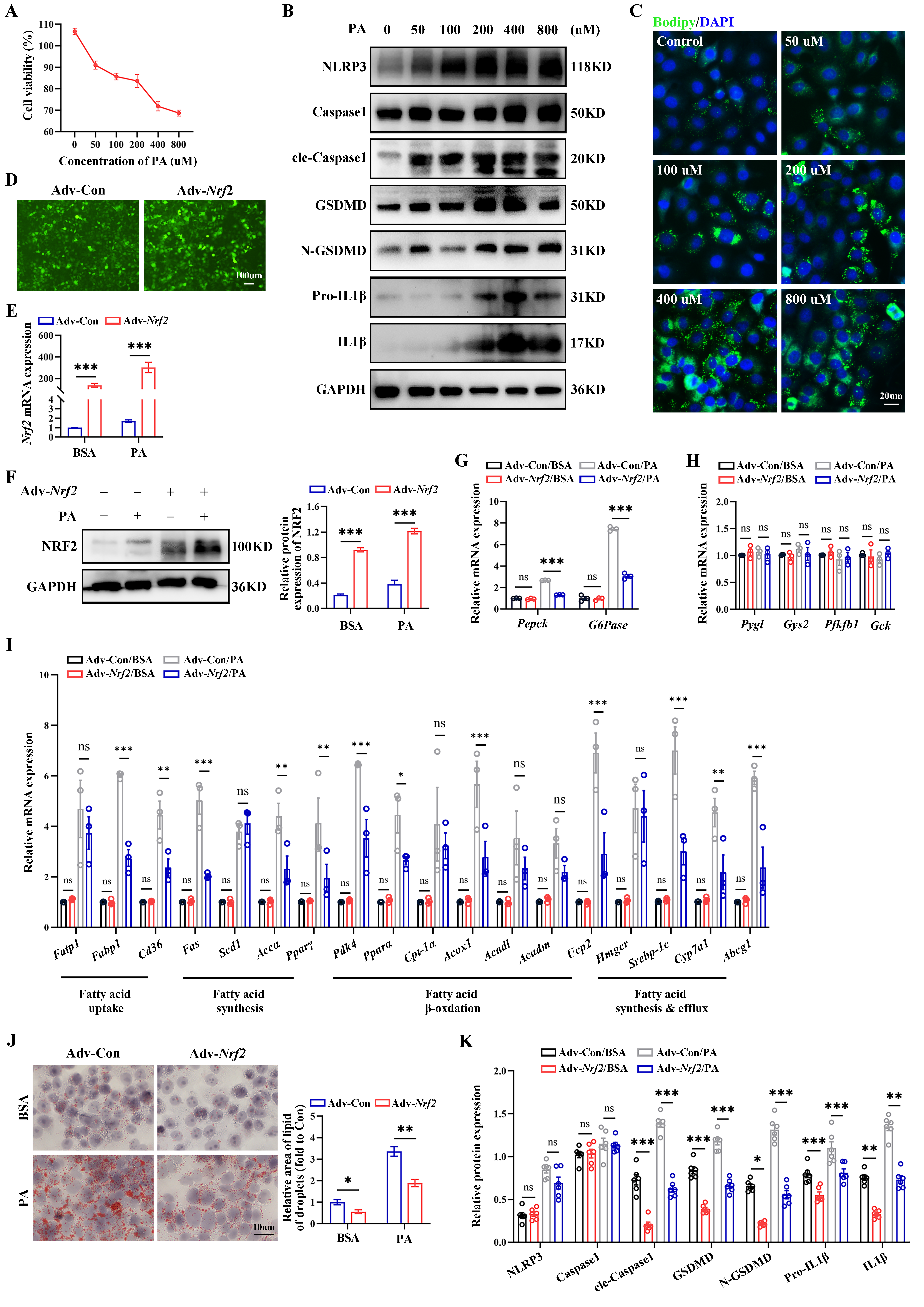

Supplement: Supplementary file 6 — Supporting Information [file CTM2-15-e70233-s001.tif]

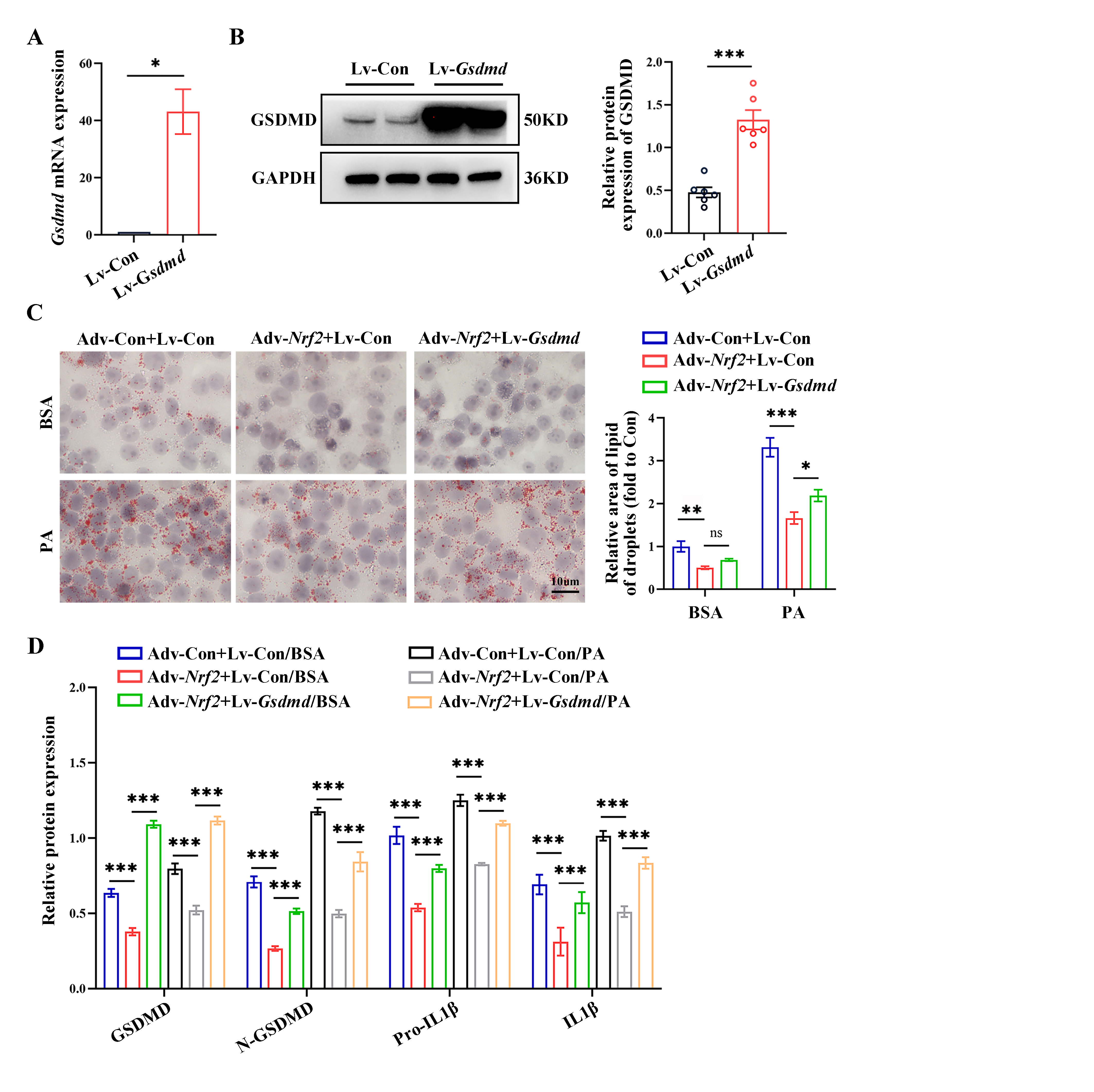

Supplement: Supplementary file 7 — Supporting Information [file CTM2-15-e70233-s008.tif]

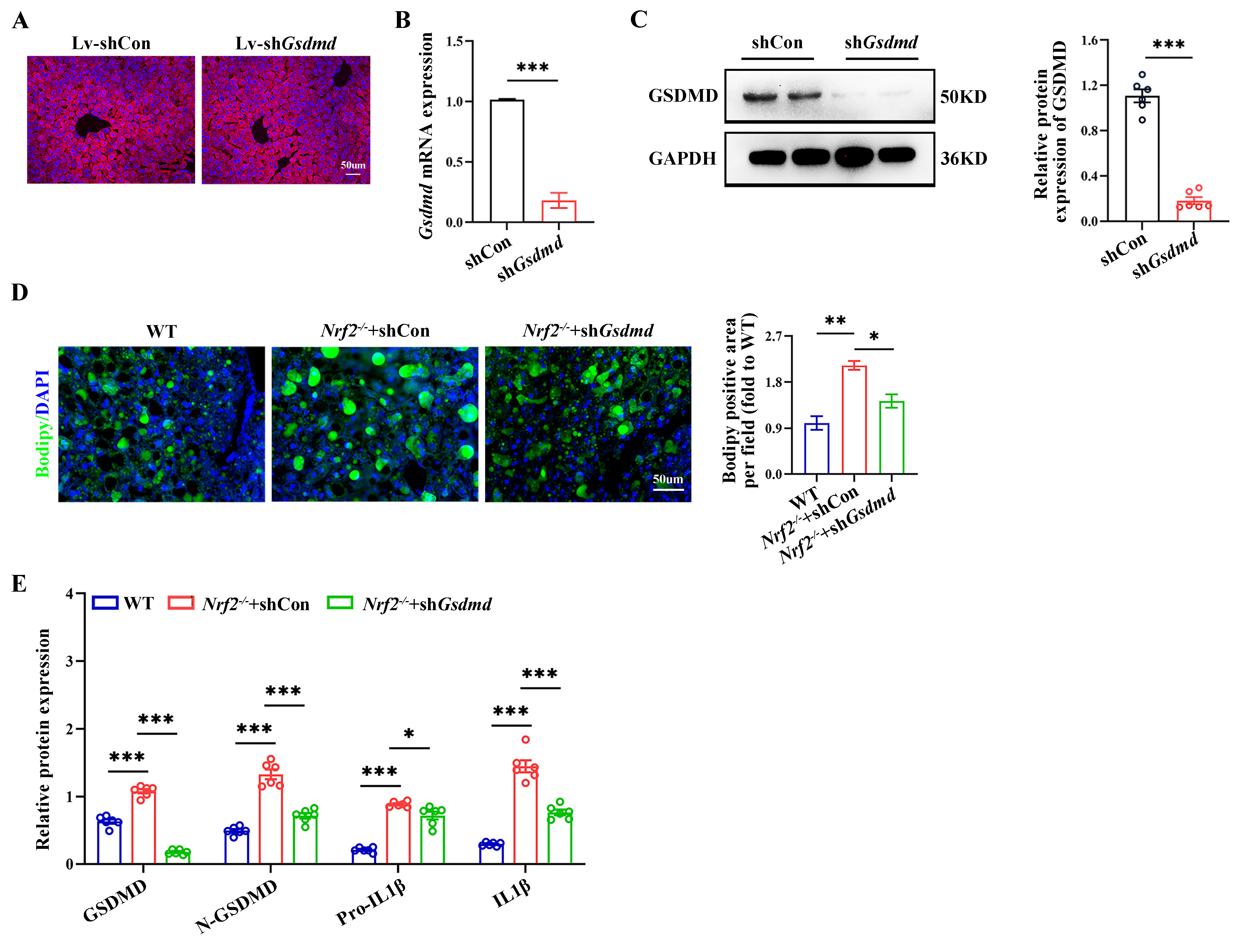

Supplement: Supplementary file 8 — Supporting Information [file CTM2-15-e70233-s007.tiff]
