# Supplementary material for: Nuclear factor erythroid 2‐related factor 2 ameliorates disordered glucose and lipid metabolism in liver: Involvement of gasdermin D in regulating pyroptosis
Source: Clin Transl Med. 2025 Feb 24;15(3):e70233. doi: 10.1002/ctm2.70233 (PMC11850759; doi:10.1002/ctm2.70233)
Supplement: Supplementary file 9 — Supporting Information [file CTM2-15-e70233-s002.docx]

| **Table S1. Antibodies** | |  |  |
| --- | --- | --- | --- |
| **REAGENT or RESOURCE** | **SOURCE** | **Application and dilution ratio** | **IDENTIFIER** |
| anti-NRF2 | Abcam, UK | WB (1:1000) | ab62352 |
| anti-NRF2 | Novus, USA | IF (1:200), WB (1:1000) | NBP1-32822 |
| anti-Albumin | Santa Cruz Biotechnology, USA | IF (1:100) | sc-271605 |
| anti-Cytokeratin 19 | Santa Cruz Biotechnology, USA | IF (1:100) | sc-376126 |
| anti-CD31 | Santa Cruz Biotechnology, USA | IF (1:100) | sc-20071 |
| Anti-Desmin | Santa Cruz Biotechnology, USA | IF (1:100) | sc-65983 |
| anti-NLRP3 | Abcam, UK | WB (1:1000) | ab263899 |
| anti-Caspase1 | Abcam, UK | WB (1:1000) | ab207802 |
| anti-Caspase11 p20 | Santa Cruz Biotechnology, USA | WB (1:500) | sc-374615 |
| anti-cle-Caspase1 | CST, USA | WB (1:1000) | 4199 |
| anti-cle-Caspase1 | Santa Cruz Biotechnology, USA | IF (1:100) | sc-398715 |
| anti-GSDMD | Abcam, UK | WB (1:1000) | ab210070 |
| anti-GSDMD | Novus, USA | IF (1:200) | NBP2-33422 |
| anti-N-GSDMD | Abcam, UK | WB (1:1000) | ab215203 |
| anti-IL1β | Abcam, UK | IF (1:200), WB (1:1000) | ab216995 |
| anti-F4/80 | Abcam, UK | IF (1:200), WB (1:1000) | ab90247 |
| Anti-LminB1 | Hua-bio, China | WB (1:1000) | ET1606-27 |
| anti-GAPDH | Hua-bio, China | WB (1:1000) | ET1601-4 |
| Goat Anti-Rabbit IgG (H+L) | Beyotime, China | WB (1:1000) | A0208 |
| Goat Anti-Mouse IgG (H+L) | Beyotime, China | WB (1:1000) | A0216 |
| Goat Anti-Mouse IgG H&L (Alexa Fluor® 555) | Abcam, UK | IF (1:200) | ab150114 |
| Goat Anti-Rabbit IgG H&L (Alexa Fluor® 488) | Abcam, UK | IF (1:200) | ab150077 |

**Table S2. Sequences of qPCR primers used in this study**

| **Gene name** | **Forward primer (Sequence: 5’-3’)** | **Reverse primer (Sequence: 5’-3’)** |
| --- | --- | --- |
| **Mouse** |  |  |
| *Actin* | CGTGCGTGACATCAAAGAGAAG | CAAGAAGGAAGGCTGGAAAAGA |
| *Nrf2* | TAGATGACCATGAGTCGCTTGC | GCCAAACTTGCTCCATGTCC |
| *G6pase* | CGACTCGCTATCTCCAAGTGA | GTTGAACCAGTCTCCGACCA |
| *Pepck* | CTGCATAACGGTCTGGACTTC | GCCTTCCACGAACTTCCTCAC |
| *Pygl* | GAGAAGCGACGGCAGATCAG | CTTGACCAGAGTGAAGTGCAG |
| *Gys2* | ACCAAGGCCAAAACGACAG | GGGCTCACATTGTTCTACTTGA |
| *Pfkfb1* | ATGAGCTGCCCTATCTCAAGT | GTCCCGGTGTGTGTTCACAG |
| *Gck* | AGGAGGCCAGTGTAAAGATGT | CTCCCAGGTCTAAGGAGAGAAA |
| *Fatp1* | CTGGGACTTCCGTGGACCT | TCTTGCAGACGATACGCAGAA |
| *Fabp1* | TGGTCCGCAATGAGTTCACCCT | CCAGCTTGACGACTGCCTTGACTT |
| *Cd36* | ATGGGCTGTGATCGGAACTG | TTTGCCACGTCATCTGGGTTT |
| *Fas* | CTGCGGAAACTTCAGGAAATG | GGTTCGGAATGCTATCCAGG |
| *Scd1* | TCTTCCTTATCATTGCCAACACCA | GCGTTGAGCACCAGAGTGTATCG |
| *Accα* | GGCCAGTGCTATGCTGAGAT | AGGGTCAAGTGCTGCTCCA |
| *Pparγ* | ATTCTGGCCCACCAACTTCGG | TGGAAGCCTGATGCTTTATCCCCA |
| *Pdk4* | TTCACACCTTCACCACATGC | AAAGGGCGGTTTTCTTGATG |
| *Pparα* | AACATCGAGTGTCGAATATGTGG | CCGAATAGTTCGCCGAAAGAA |
| *Cpt1α* | CTCCGCCTGAGCCATGAAG | CACCAGTGATGATGCCATTCT |
| *Acox1* | TAACTTCCTCACTCGAAGCCA | AGTTCCATGACCCATCTCTGTC |
| *Acadl* | GGAGTAAGAACGAACGCCAA | GCCACGACGATCACGAGAT |
| *Acadm* | TGGCGTATGGGTGTACAGGG | CCAAATACTTCTTTTTTTGTTGATCA |
| *Ucp2* | GCTGGTGGTGGTCGGAGATA | ACTGGCCCAAGGCAGAGTT |
| *Hmgcr* | ATCATGTGCTGCTTCGGCTGCAT | AAATTGGACGACCCTCACGGCT |
| *Srebp1c* | CACTTCTGGAGACATCGCAAAC | ATGGTAGACAACAGCCGCATC |
| *Cyp7a1* | TCAAAGAGCGCTGTCTGGGTCA | TTTCCCGGGCTTTATGTGCGGT |
| *Abcg1* | TGAACCCGTTTCTTTGGCACCG | AGTCCCGCATGATGCTGAGGAA |
| *Nlrp3* | ATCAACAGGCGAGACCTCTG | GTCCTCCTGGCATACCATAGA |
| *Caspase1* | ACAAGGCACGGGACCTATG | TCCCAGTCAGTCCTGGAAATG |
| *Gsdmd* | CCATCGGCCTTTGAGAAAGTG | ACACATGAATAACGGGGTTTCC |
| *IL1β* | GAAATGCCACCTTTTGACAGTG | TGGATGCTCTCATCAGGACAG |
| *IL18* | GACTCTTGCGTCAACTTCAAGG | CAGGCTGTCTTTTGTCAACGA |
| **Human** | | |
| *Gapdh* | GGAGCGAGATCCCTCCAAAAT | GGCTGTTGTCATACTTCTCATGG |
| *Nrf2* | TCCAGTCAGAAACCAGTGGAT | GAATGTCTGCGCCAAAAGCTG |
| *G6pase* | TCAAGCAGTCCTCCCACCCTAC | AGCCAAGATCGTGCCACTCCA |
| *Pepck* | GCCCTGGGAGATGGTGACTTTG | ATAGCCGCTGCCGAAGGAGAT |
| *Pygl* | TGCCCGGCTACATGAATAACA | TGTCATTGGGATAGAGGACCC |
| *Gys2* | GTGGAACAGTGTGAACCTGTAA | AGGACTTCCTTCTATCAGCCAT |
| *Pfkfb1* | GGCCAGTATCGACGAGAGG | CAAAAACCGCAACATGACCTTC |
| *Gck* | GCAGAAGGGAACAATGTCGTG | CGTAGTAGCAGGAGATCATCGT |
| *Fatp1* | GGGGCAGTGTCTCATCTATGG | CCGATGTACTGAACCACCGT |
| *Fabp1* | ATGAGTTTCTCCGGCAAGTACC | CTCTTCCGGCAGACCGATTG |
| *Cd36* | AGTTGGAGACC TGC TTATC | CTTGAATGTTGC TGC TGTT |
| *Fas* | GTCCACCAGCAACATCAG | TTCTCCAGCAAGCCATCT |
| *Scd1* | CGATATGCTGTGGTGCTTA | AAGGAGTGGTGGTAGTTGT |
| *Acc1* | ATGTCTGGCTTGCACCTAGTA | CCCCAAAGCGAGTAACAAATTCT |
| *Pparγ* | ACTCCACATTACGAAGACAT | CTCCACAGACACGACATT |
| *Pdk4* | GGAGCATTTCTCGCGCTACA | ACAGGCAATTCTTGTCGCAAA |
| *Pparα* | GCGAGGATAGTTC TGGAAG | AGGATAAGTC ACCGAGGAG |
| *Cpt1α* | TCCAGTTGGCTTATCGTGGTG | TCCAGAGTCCGATTGATTTTTGC |
| *Acox1* | ACTCGCAGCCAGCGTTATG | AGGGTCAGCGATGCCAAAC |
| *Acadl* | TGCAATAGCAATGACAGAGCC | CGCAACTACAATCACAACATCAC |
| *Acadm* | TCCTTCTTCTAACTGGTTCC | ACACATCAATGGCTCCTC |
| *Ucp2* | CCCCGAAGCCTCTACAATGG | CTGAGCTTGGAATCGGACCTT |
| *Hmgcr* | TGATTGACCTTTCCAGAGCAAG | CTAAAATTGCCATTCCACGAGC |
| *Srebp1c* | GGAGCCATGGATTGCACTTT | TCAAATAGGCCAGGGAAGTCA |
| *Cyp7a1* | GAGAAGGCAAACGGGTGAAC | GGATTGGCACCAAATTGCAGA |
| *Abcg1* | ATTCAGGGACCTTTCCTATTCGG | CTCACCACTATTGAACTTCCCG |
| *Nlrp3* | CGTGAGTCCCATTAAGATGGAGT | CCCGACAGTGGATATAGAACAGA |
| *Caspase1* | CGCACACGTCTTGCTCTCATT | TTTTCACATCTACGCTGTACCCC |
| *Gsdmd* | GAGTGTGGCCTAGAGCTGG | GGCTCAGTCCTGATAGCAGTG |
| *IL1β* | TCCCCAGCCCTTTTGTTGA | TTAGAACCAAATGTGCCCGTG |
| *IL18* | AAACCTGGAATCAGATTACTTTGGC | ATAAATATGGTCCGGGGTGCATT |
| **Genotyping Primers** | | |
| *Nrf2-F* | GCCTGAGAGCTGTAGGCCC |  |
| *Nrf2-WT-R* | GGAATGGAAAATAGCTCCTGCC |  |
| *Nrf2-KO-R* | GACAGTATCGGCCTCAGGAA |  |
| *Nrf2-flox-F* | AGGCCTGTAAACTACAAGTCCAT |  |
| *Nrf2-flox-R* | ACTCCATGTATTTAAAGGCAGACTTC |  |
|  |  |  |

| Number | N1301 | N1302 | N1303 | N1304 | F1301 | F1302 | F1303 | F1304 |
| --- | --- | --- | --- | --- | --- | --- | --- | --- |
| Group | Normal control | Normal control | Normal control | Normal control | MAFLD | MAFLD | MAFLD | MAFLD |
| Sex | female | female | male | male | female | female | male | male |
| Age | 45.00 | 34.00 | 66.00 | 83.00 | 71.00 | 53.00 | 60.00 | 36.00 |
| Waist circumference (cm) | 81.00 | 78.00 | 85.00 | 89.00 | 101.00 | 93.00 | 96.00 | 96.00 |
| Hip circumference (cm) | 90.00 | 92.00 | 98.00 | 99.00 | 103.00 | 96.00 | 96.00 | 99.00 |
| WHR | 0.90 | 0.85 | 0.87 | 0.90 | 0.98 | 0.97 | 1.00 | 0.97 |
| Body weight (Kg) | 51 | 48 | 50 | 60 | 62 | 74 | 65 | 80 |
| Height (cm) | 152 | 162 | 163 | 170 | 154 | 161 | 159 | 175 |
| BMI | 22.07 | 18.29 | 18.82 | 20.76 | 26.14 | 28.55 | 25.71 | 26.12 |
| Complication | No | No | No | No | Hyperte-ntion | No | No | No |
| Alcohol abuse | No | No | No | No | No | No | No | No |
| Hepatitis virus | No | No | No | No | No | No | No | No |
| Total bilirubin(umol/L) | 10.00 | 13.70 | 5.30 | 13.30 | 10.40 | 13.20 | 26.70 | 62.20 |
| ALT(U/L) | 73.00 | 18.00 | 44.00 | 52.00 | 38.00 | 47.00 | 70.00 | 127.00 |
| AST(U/L) | 29.00 | 19.00 | 37.00 | 55.00 | 28.00 | 33.00 | 47.00 | 85.00 |
| ALP(U/L) | 63.00 | 72.00 | 81.00 | 78.00 | 109.00 | 59.00 | 64.00 | 268.00 |
| γ-GT(U/L) | 65.00 | 14.00 | 91.00 | 55.00 | 116.00 | 61.00 | 147.00 | 491.00 |
| Albumin(g/L) | 41.00 | 43.00 | 36.00 | 32.00 | 40.00 | 43.00 | 33.00 | 42.00 |
| TG(mmol/l) | 0.69 | 0.65 | 0.61 | 0.63 | 1.38 | 1.30 | 1.48 | 2.40 |
| TC(mmol/l) | 2.67 | 3.52 | 3.51 | 2.44 | 4.49 | 5.45 | 4.97 | 6.57 |
| HDL(mmol/l) | 1.56 | 1.20 | 1.49 | 0.93 | 0.82 | 3.63 | 1.48 | 0.22 |
| LDL(mmol/l) | 1.00 | 2.25 | 1.91 | 1.07 | 3.26 | 1.47 | 3.37 | 2.63 |

**Table S3. Clinical parameters in MAFLD patients**

**Table S4. Prediction of NRF2’s binding motifs of the promoters in pyroptosis-related genes.**

|  | **Score** | **Relative score** | **Start** | **End** | **Strand** | **Predicted sequence** | **p-value** | **q-value** |
| --- | --- | --- | --- | --- | --- | --- | --- | --- |
| **Caspase4** | 11.93825 | 0.898435 | 1886 | 1896 | + | atgacaaggca | 2.08E-05 | 0.0847 |
|  | 11.08737 | 0.877286 | 688 | 698 | - | ataacacagca |  |  |
| **Caspase5** | 10.15562 | 0.854127 | 1797 | 1807 | - | atgacaaaaca |  |  |
|  | 10.15562 | 0.854127 | 2630 | 2640 | - | atgacaaaaca |  |  |
|  | 9.503193 | 0.83791 | 1222 | 1232 | + | gttacacagcc | 8.91E-05 | 0.355 |
|  | 9.503193 | 0.83791 | 2055 | 2065 | + | gttacacagcc |  |  |
|  | 8.537298 | 0.813902 | 741 | 751 | + | attcctcagca |  |  |
| **Caspase8** | 12.72875 | 0.918084 | 668 | 678 | - | gtgacagagca | 1.16E-05 | 0.113 |
|  | 11.64902 | 0.891246 | 1939 | 1949 | + | gtgactcagga |  |  |
|  | 8.143593 | 0.804117 | 1892 | 1902 | - | ctgagtgagca |  |  |
|  | 8.072969 | 0.802361 | 1783 | 1793 | - | gtgactttgga |  |  |
| **GSDMC** | 11.52727 | 0.88822 | 100 | 110 | + | atgatccagca | 3.00E-05 | 0.157 |
| **NLRP1** | 9.609231 | 0.840546 | 1348 | 1358 | + | atgagtaggca | 9.52E-05 | 0.242 |
|  | 9.310809 | 0.833129 | 1355 | 1365 | - | atgacattgcc | 9.72E-05 | 0.242 |
|  | 8.659159 | 0.816931 | 1835 | 1845 | - | ctgacgctgca |  |  |
| **NLRP3** | 10.88141 | 0.872167 | 2301 | 2311 | - | GCGACACAGCA |  |  |
|  | 9.890355 | 0.847534 | 1098 | 1108 | - | ttgaccaagca |  |  |
|  | 9.303911 | 0.832957 | 1620 | 1630 | - | ctgacccagcc | 8.56E-05 | 0.47 |
|  | 8.424603 | 0.811101 | 509 | 519 | - | gtgccactgca |  |  |
|  | 8.248538 | 0.806725 | 1889 | 1899 | + | gtgattaacca |  |  |
|  | 8.037945 | 0.801491 | 1712 | 1722 | - | ctgacactgcc |  |  |
